# Shallow recurrent decoders for neural and behavioural dynamics

**DOI:** 10.1098/rstb.2024.0461

**Published:** 2026-09-17

**Authors:** Amy Rude, J. Nathan Kutz

**Affiliations:** ^1^ Department of Applied Mathematics, University of Washington, Seattle, WA, USA; ^2^ Autodesk Research, London, UK

**Keywords:** computational neuroscience, machine learning, population codes, shallow recurrent decoding, sensing

## Abstract

Machine learning algorithms are affording new opportunities for building bio-inspired and data-driven models characterizing neural activity. Critical to understanding decision-making and behaviour is quantifying the relationship between the activity of neuronal population codes and individual neurons. We leverage a SHallow REcurrent Decoder (SHRED) architecture for mapping the dynamics of population codes to individual neurons and other proxy measures of neural activity and behaviour. SHRED is constructed from a temporal sequence model, which encodes the temporal dynamics of limited sensor data in multiple scenarios, and a shallow decoder, which reconstructs the corresponding high-dimensional neuronal and/or behavioural states. It is a robust and flexible sensing strategy which allows for decoding the diversity of neural measurements with only a few sensor measurements. Thus, estimates of whole-brain activity, behaviour and individual neurons can be constructed with only a few neural time-series recordings. Several examples in this article further highlight the potential of leveraging non-invasive or minimally invasive measurements to estimate large-scale brain dynamics. We empirically demonstrate the capabilities of the method on a number of model organisms including *Caenorhabditis elegans*, mouse, zebrafish and human biolocomotion.

This article is part of the discussion meeting issue ‘Digital healthcare for the management of functional neurological disorders’.

## 1. Introduction

High-dimensional networked biological systems are ubiquitous and characterized by a large connectivity graph whose structure determines how the system operates as a whole [1,2]. Typically the connectivity is so complex that the functionality, control and robustness of the network of interest are impossible to characterize using currently available methods. Moreover, with few exceptions, underlying nonlinearities impair our ability to construct analytically tractable solutions, forcing one to rely on experiments and/or modern high-performance computing to study a given system. However, advances in experimental and computational neuroscience over the past decades have revealed two critical, and seemingly ubiquitous, phenomena: (i) that the nervous system leverages dimensionality reduction when it encodes behaviour and decision-making [3–6], and (ii) that sparsity is fundamental in encoding and decoding information in the network [7–9]. Thus, instead of relying on individual neurons, one can consider the dynamic encoding space, or low-dimensional manifold [10], of the brain for representing complex decision-making and cognitive capabilities. A full understanding of the computational process encoded throughout a nervous system must reconcile these two key observations that transform sensory input into decision-making and motor representations. These two observations are the basis of our proposed SHallow REcurrent Decoder (SHRED), a deep learning architecture that allows for an efficient and robust mathematical framework connecting the activity of individual (sparse) neurons to population codes and behavioural measurements. Thus the monitoring of a small number of neurons or their proxies is capable of reconstructing brain-wide activity or proxy behavioural outcomes, as we demonstrate in the nematode *Caenorhabditis elegans*, the mouse, zebrafish and in human biolocomotion.

Originally developed as a sensing strategy, SHRED [11] is a promising deep learning architecture with impressive reconstruction and forecasting results in the low-data limit [12–16]. In this article, we empirically demonstrate SHRED's ability to uncover population neural codes from a limited number of sensors. While SHRED has traditionally been applied to problems in physics and engineering, the dynamic, high-dimensional and coupled nature of neural population codes makes neuroscience an ideal domain for this architecture. Importantly, SHRED's basic architecture connects sparse sampling of signals to population codes for applications in neuroscience. SHRED leverages the mathematical theory of Takens’ embedding theorem [17], which shows that time-series measurements of a single state variable dynamically coupled to the system are sufficient to reconstruct a state space diffeomorphic to the true dynamics. In the context of neuroscience and supervised machine learning, this implies that recordings from a single neuron could, in principle, be sufficient to recover population activity. Previous work has shown SHRED can achieve excellent performance in sensing and reduced order modelling examples ranging from weather and atmospheric forecasting [11], plasma physics [12], nuclear reactors [14] and turbulent flow reconstructions [15]. Theoretically rooted in the classic partial differential equation (PDE) method of separation of variables [11,15], the decoding-only strategy of SHRED circumvents the computation of inverse pairs, i.e. an encoder and the corresponding decoder. It has been well-known for decades that the computation of the inverse of a matrix is highly unstable and ill-posed [18,19]. The root of the ill-posedness can be understood from the singular value decomposition whose singular values $\sigma_{j}$ become $1/\sigma_{j}$ in the inverse matrix decomposition. For singular values going to zero, there arises a divide by zero issue which results in the ill-conditioning. By decoding only, SHRED avoids this problem and learns a single embedding without the corresponding inversion. Moreover, the SHRED architecture provides an approximation to an operator which maps the time trajectories to full state estimates. Tomasetto *et al.* [15] compare the SHRED architecture to leading operator learning methods showing the superior and robust performance advantage.

**Figure 1.**
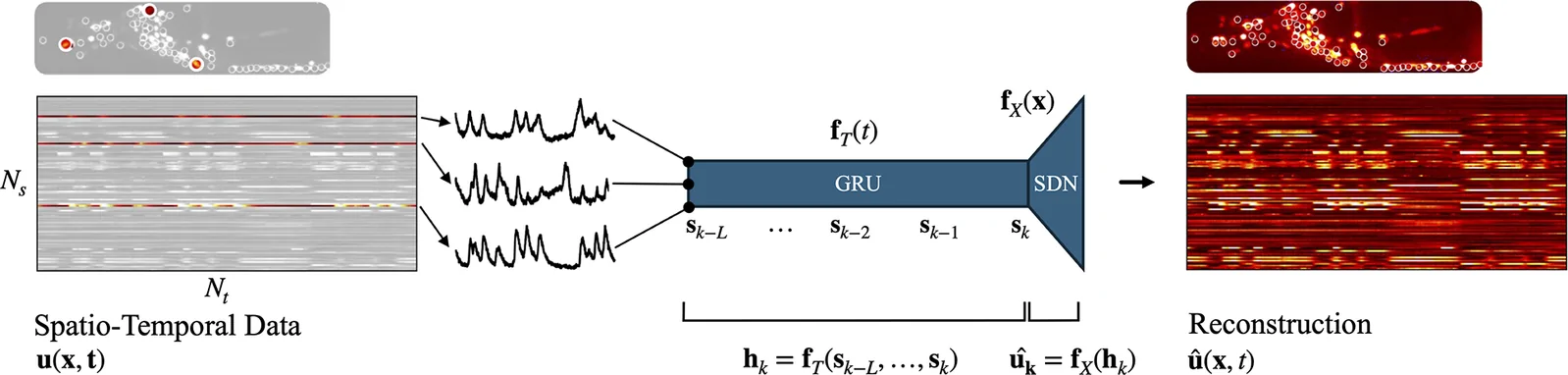
Diagram of SHallow REcurrent decoder (SHRED) architecture. Time-series data from three neurons $\{s_{i}{\}}_{k-L}^{k}$ are fed into a gated recurrent unit (GRU). The output from GRU $h_{k}$ is the input for the shallow decoder network (SDN), mapping back to the full state space. *Caenorhabditis elegans* neuron image (top) is adapted from Kato *et al.* [20].

SHRED is leveraged on a number of toy models in neuroscience, including the nematode *C. elegans*, the mouse, zebrafish and human biolocomotion. In each case, SHRED is shown to be robust and accurate in approximating population codes using only a limited number of neurons. Figure 1 details the basic architecture of the neural network, where the latent space of a temporal encoder is used to reconstruct full activity across neurons in *C. elegans*. An extensive study is made for each model system showing that the architecture can be used to critically reconstruct population codes from only a highly limited number of individual neuron recordings. In the case of human biolocomotion, it is used instead to estimate gait motion using only a single sensor (smart watch inertial measurement unit (IMU)). The architecture is thus promising for extracting critical information on decision-making and motor commands with only limited, and potentially non-invasive, measurements. This provides a valuable potential reduction to practice for digital healthcare for the management of functional neurological disorders by encoding critical metrics of cognition through proxy measurements encoded through SHRED.

## 2. Mathematical background for SHRED

SHRED consists of a combination of recurrence and decoding, with the former operation capturing the temporal behaviour of limited sensor data and the latter operation performing a spatial upscaling to recover the corresponding high-dimensional state. A similar split in spatial and temporal components is taken into account by the well-known method of separation of variables for solving linear PDEs [21], which assumes that the solution has the form u(x,t)=T(t)X(x), with T(t) governing the temporal dynamics and X(x) the spatial dynamics. Indeed, it has been shown that in the limit of linear dynamics with constant coefficients, the SHRED architecture has a closed form analytic solution [11,15]. Specifically, for linear PDEs, the solution is given by a sum of modes with exponential time dynamics. Indeed, in the linear limit, the analytic solution of the dynamic mode decomposition [22,23] is a sum of modes with exponential time dynamics. Of note is that the architecture can be used to produce estimates for uncertainty quantification [24] along with multi-scale features [25].

Thus SHRED is a generalization of separation of variables with nonlinear (neural network) approximations. A compact representation of the SHRED algorithm, as shown in figure 1, is as two neural networks jointly trained to the form

(2.1) $$A=X(x)T(t)\to A=f_{\theta_{1}}(x)\circ g_{\theta_{2}}(t),$$

where A is a data matrix whose columns are time samples and whose rows are the full state space, $g_{\theta_{2}}(t)$ is trained largely to encode sequential (time) data and $f_{\theta_{1}}(x)$ is largely responsible for mapping from the latent space of the sequential encoding to the full state space. Of course, the time and space encodings are not independent as they are jointly trained, and the decoder is dependent on the time history of the sequence model.

To be more precise, as a deep learning architecture, SHRED aims to reconstruct high-dimensional neuronal data from $N_{s}$ individual neural recordings. Instead of learning the one-shot reconstruction map $s_{k}=\{u(x_{s},t_{k}){\}}_{s=1}^{N_{s}}\to u_{k}$ for $k=1,\ldots ,N_{t}$—as considered by, e.g. [26–29]—we take advantage of the temporal history of the individual neuron values. Specifically, a recurrent neural network $f_{T}$ encodes the neuron measurements over a time window of length $L\leq N_{t}$ into a latent representation of dimension $N_{l}$, that is

$$\begin{array}{r} f_{T}:\underset{L\text{ times }}{\underbrace{R^{N_{s}}\times \ldots \times R^{N_{s}}}}\to R^{N_{l}},\\ h_{k}=f_{T}(s_{k-L},\ldots ,s_{k}), \end{array}$$

where the hyperparameter L identifies the number of lags considered by SHRED, and may be selected according to the problem-specific evolution rate or periodicity. The high-dimensional state is then approximated through a decoder $f_{X}$, which performs a nonlinear projection of the latent representation onto the state space

$$\begin{array}{l} f_{X}:R^{N_{l}}\to R^{N_{h}},\\ u_{k}\approx {\hat{u}}_{k}=f_{X}(h_{k})=f_{X}(f_{T}(s_{k-L},\ldots ,s_{k})). \end{array}$$

In this work, we consider a gated recurrent unit (GRU) network [30] to model the temporal dependency of sensor data, and a shallow decoder network (SDN) as the latent-to-state map. SHRED is a modular framework, allowing for various forms of temporal encoding and spatial decoding, including convolutional neural networks [31], long short-term memory [32], echo state networks [33], transformers [34] and variational autoencoders [35]. Importantly, the decoding-only strategy circumvents learning an encoder–decoder pair [36] which leads to numerical stiffness [18,19].

## 3. Model systems

The SHRED architecture maps limited, time-series sensor measurements to the full, high-dimensional neural or behaviour data. In what follows, we show how measuring only a limited number of neurons or sensors can be sufficient to estimate the full state space across four model systems: *C. elegans* ${\text{Ca}}^{2+}$ imaging data (§3a), mouse local field potential (LFP) and spiking activity (§3b), zebrafish forebrain video recordings (§3c) and human locomotion data (§3e). Together, these applications illustrate the versatility of our algorithm in addressing diverse and challenging tasks. Details of hyperparameter tuning and parameter selection can be found in appendix A. For details regarding the dataset and access to the code used to produce the results, see data accessibility. Table 1 gives a comprehensive summary of all mean squared errors (m.s.e.) and §3d details a baseline comparison of SHRED to a linear model for §3a–c.

**Table 1.** Mean square error (m.s.e.) for all experiments presented in §3a, b and c. DG, drifting grating stimulus; FL, flash stimulus; FR, firing rate; LFP, local field potential; asterisk, data for input into the model. Rows separated by horizontal lines represent separate models trained for the corresponding dataset.

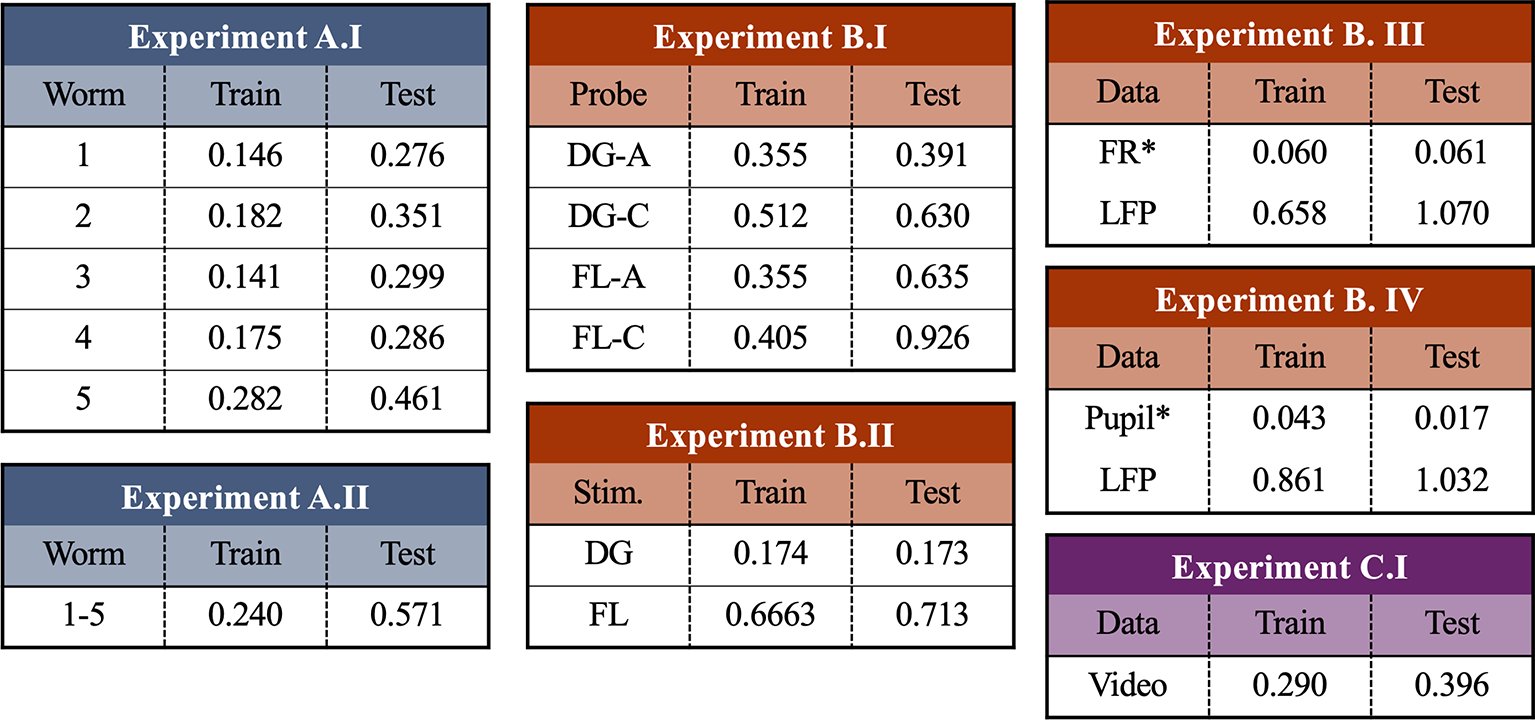

### (a) *Caenorhabditis elegans* nematode

The nematode (*C. elegans*) is a perfect model organism to consider in the context of neurosensory integration as it comprises only 302 sensory, motor and interneurons whose electro-physical connections (i.e. its connectome) are known and stereotyped [37,38]. In addition, it possesses only a small number of sensory neurons, often linked to specific stimuli [39]; neural activity can be imaged in real time; and its range of behavioural responses, during chemotaxis for instance, are varied yet limited, approximately confined to five observable motor states that are related to forward and backward crawling, omega turns, head sweeps and brief pause states. Thus it is reasonable to posit a complete model of its neurosensory integration capabilities. Aiding in this effort is the near-complete connectivity data for the gap junctions and chemical synapses connecting the sensory neurons to the inter- and motor neurons [40]. With access to a complete record of sensory neuron activity across the sensory periphery as well as a complete record of command motor neuron activity and locomotor behaviour, we are well poised to understand how decision-making circuits collapse sensory representations into the most basic commands that shape behaviour by regulating muscle activity. Recent experiments in behaving animals retain proprioceptive feedback features which strongly shape computational pathways in sensorimotor circuits [41,42].

We obtained whole-brain calcium imaging recordings of immobilized nematodes (*C. elegans*) from a publicly available dataset provided in Kato *et al.* [20]. This dataset contains data for five worms with recordings from 107 to 131 identified neurons in an environmentally controlled condition. Recordings were made for a duration of roughly 18 minutes at an average rate of 2.85 Hz. Since bleaching causes damping of ${\text{Ca}}^{2+}$ imaging signals over time, we truncated the data to only include the first half of each session [20]. In the sections that follow, we apply SHRED to this dataset for individual (§3a(i)) and cross-worm predictions (§3a(ii)). The results presented in these sections demonstrate the capabilities of SHRED to decode population-wide dynamics from sparse neural measurements. Sensitivity analysis for the model is presented in §3a(iii).

#### (i) Shred reconstruction of *Caenorhabditis elegans* neural activity

**Figure 2.**
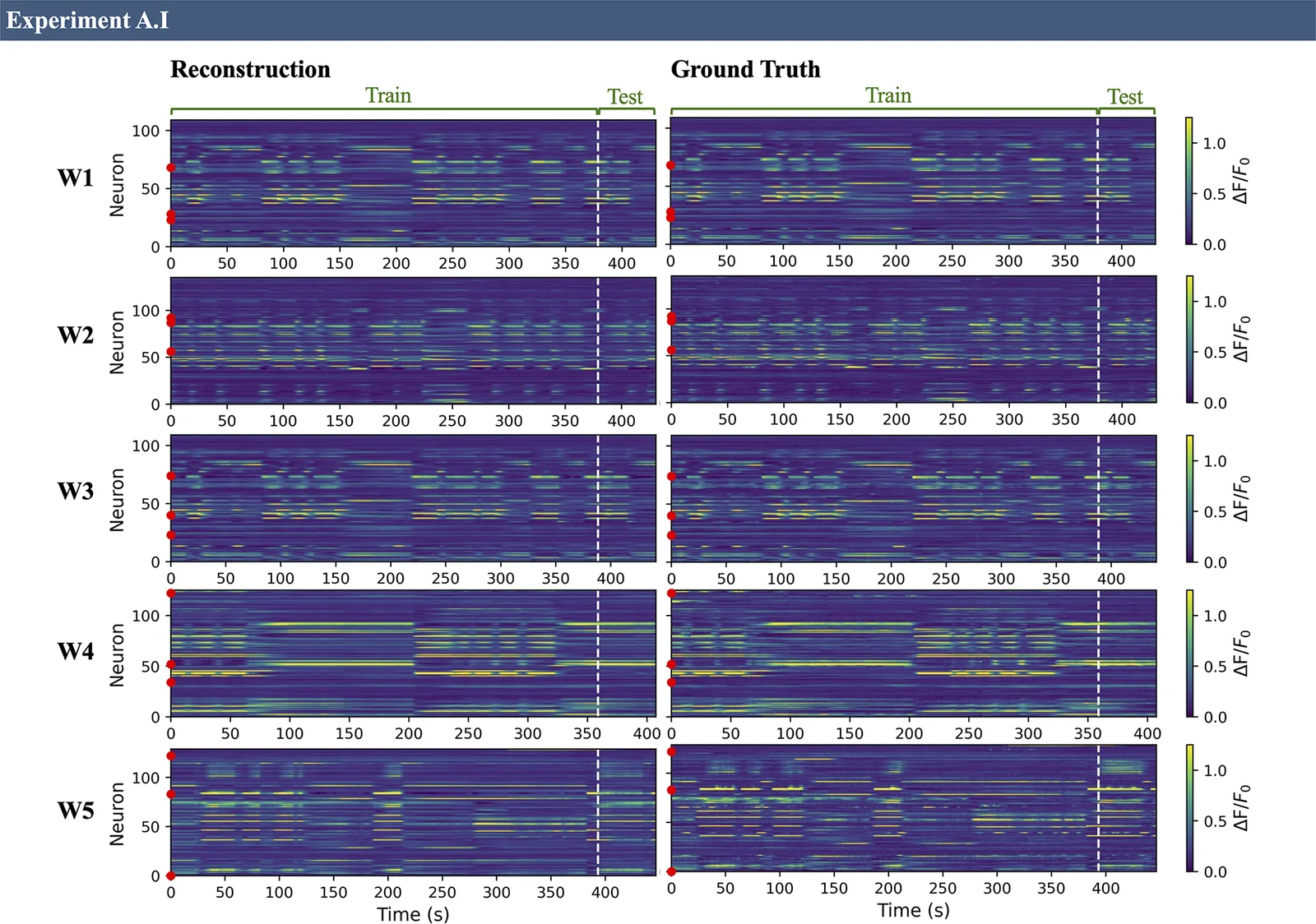
SHRED can accurately reconstruct population-wide activity from only three high variance (HV) neurons. SHRED reconstruction (left) of neural activity (right) for all five worms. General activity patterns are preserved in the reconstruction of both training and testing datasets. Red dots on the *y*-axis indicate sensors selected as input. Refer to table 1 Experiment A.I for error metrics.

We reconstructed whole-population activity for all five worms using SHRED. As shown in the carpet plots in figure 2, some neurons remained inactive for the entire duration of the recording. To ensure that our model performance was not negatively impacted by the selection of these inactive neurons, we created subgroups of 34–41 high variance (HV) neurons and 75–95 low variance (LV) neurons. Details of this split can be found in §3a(iii). To perform the reconstruction, we randomly selected three neurons from the HV group and the first 1500 time points (approx. 8.5 minutes) from each worm was split 80 : 10 : 10 (train : test : validation). With a lag L=100, the model was trained for 200 epochs. The corresponding normalized m.s.e. was calculated for both the training and testing datasets.

(3.1) $$\text{m.s.e.}=\frac{||f_{X}(h_{k})-u_{k}|{|}_{2}}{||u_{k}|{|}_{2}}.$$

Sensitivity analysis for the number of neurons selected and train/test split are outlined in §3a(iii). Further details on hyperparameter tuning and selecting these parameters can be found in appendix A. Finally, baseline comparisons are conducted in §3d.

SHRED does a remarkable job learning spatial and temporal patterns in data (figure 2 and table 1). To the naked eye, the reconstructions presented in figure 2 are near identical to the original data. Activity traces are reconstructed in a temporally precise fashion—the peaks and troughs in the activity of each neuron are well-preserved. Even for the withheld test dataset, the model performs remarkably well with the exception of worm 5 (W5). Several neurons exhibit heightened activity throughout the duration of the test data. This pattern in activity is sustained for a shorter duration in the reconstruction, resulting in a higher error. With requiring only three sensor recordings as input, SHRED has the potential to enable less invasive recording procedures and even recover information from low-quality sensors. Once trained on an individual worm, the model generalizes to new time points without a substantial loss in reconstruction accuracy. In the following section, we increase the difficulty by reconstructing activity from a worm that was completely withheld during training. Such cross-individual predictions are particularly valuable, as they further reduce the need for repeated invasive experiments.

#### (ii) Cross-worm predictions using SHRED

**Figure 3.**
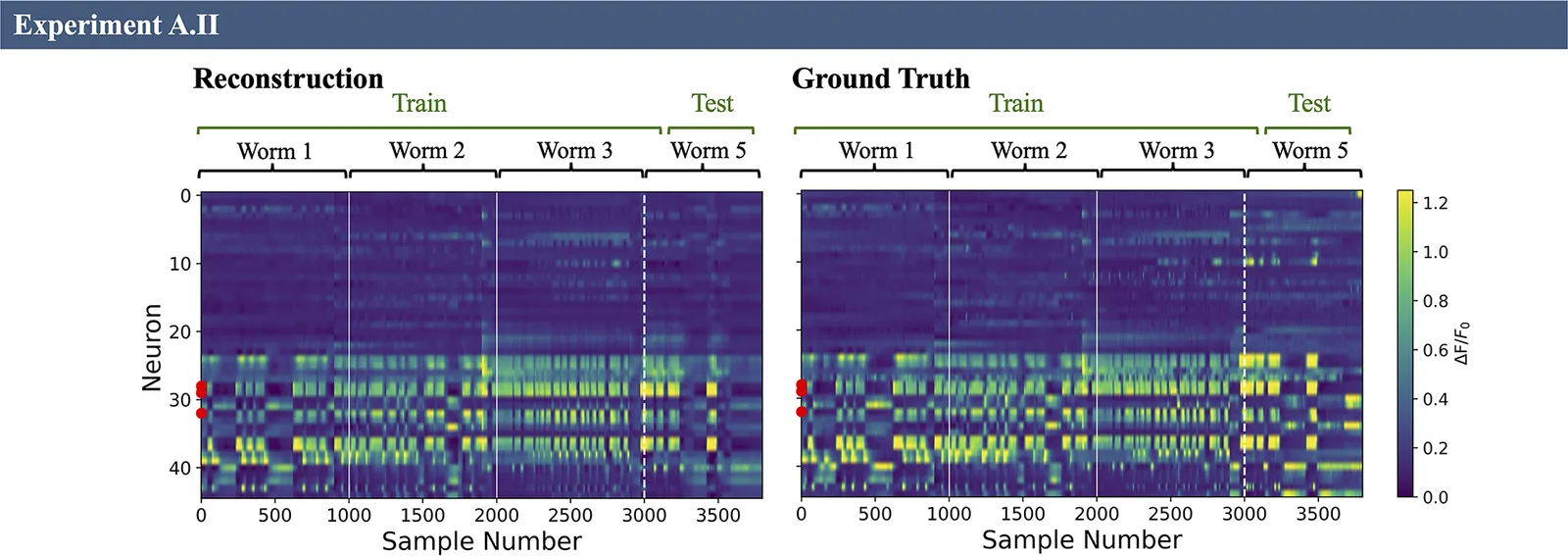
SHRED can generalize across worms. A SHRED model trained to reconstruct neural activity of worms 1–3 from three HV neurons can extrapolate to reconstruct activity for worm 5. Red dots on the* y*-axis indicate sensors selected as input. Refer to table 1 Experiment A.II for error metrics.

So far, SHRED has shown remarkable ability for reconstructing neural activity from a select number of neurons for a single individual. In this task, we leveraged the conserved patterns in activity of *C. elegans* to reconstruct neural activity across all five worms. For each worm, we selected the first 1000 time points. Only 45 neurons were recorded from across all five worms. Therefore, only these neurons were selected to be included in this trial. The training data were defined to be the first 3000 time points which correspond to the neural activity of the first three worms. The validation data were defined to be the next 1000 time points corresponding to the neural data of worm 4. The test data were defined to be the last 1000 time points which correspond to the neural data of worm 5. Reconstruction of the test dataset only includes the first 900 time points to account for the lag (L) of 100. The data were preprocessed to include 18 neurons in the HV group and 27 neurons in the LV group. We randomly selected three neurons from the HV group for the reconstruction. Results are presented in figure 3.

The training error and test error are reported in table 1A.II). As expected, since the test set is extrapolating to a new worm not included in the training, the test error is substantially higher than the training m.s.e. Nevertheless, the reconstructed activity for the fifth worm still preserves the overall trends (figure 3). Importantly, this dataset provides reliable neural measurements for five worms from the same neurons, enabling cross-individual predictions. This example illustrates the potential of SHRED to aid in minimally invasive or non-invasive recording techniques. While these datasets are challenging to obtain, we have demonstrated cross-individual testing in this section and §3e. Thus, once training is accomplished on the full dataset, it can be deployed to new subjects with access only to non-invasive sensor sets. Moreover, by successfully extrapolating beyond individuals used for training, SHRED captures distinct spatio-temporal patterns without overfitting to the training data.

#### (iii) Sensitivity analysis of SHRED performance

We also conducted some preliminary tests to study the sensitivity of the SHRED model to (i) the activity of the neuron selected, (ii) the number of neurons selected, and (iii) the data split. For these series of experiments, a limited number of neurons were selected, and SHRED was trained to predict population activity. Only the first half of the time-series data for the first *C. elegans* (W1) was used to simplify analysis.

**Figure 4.**
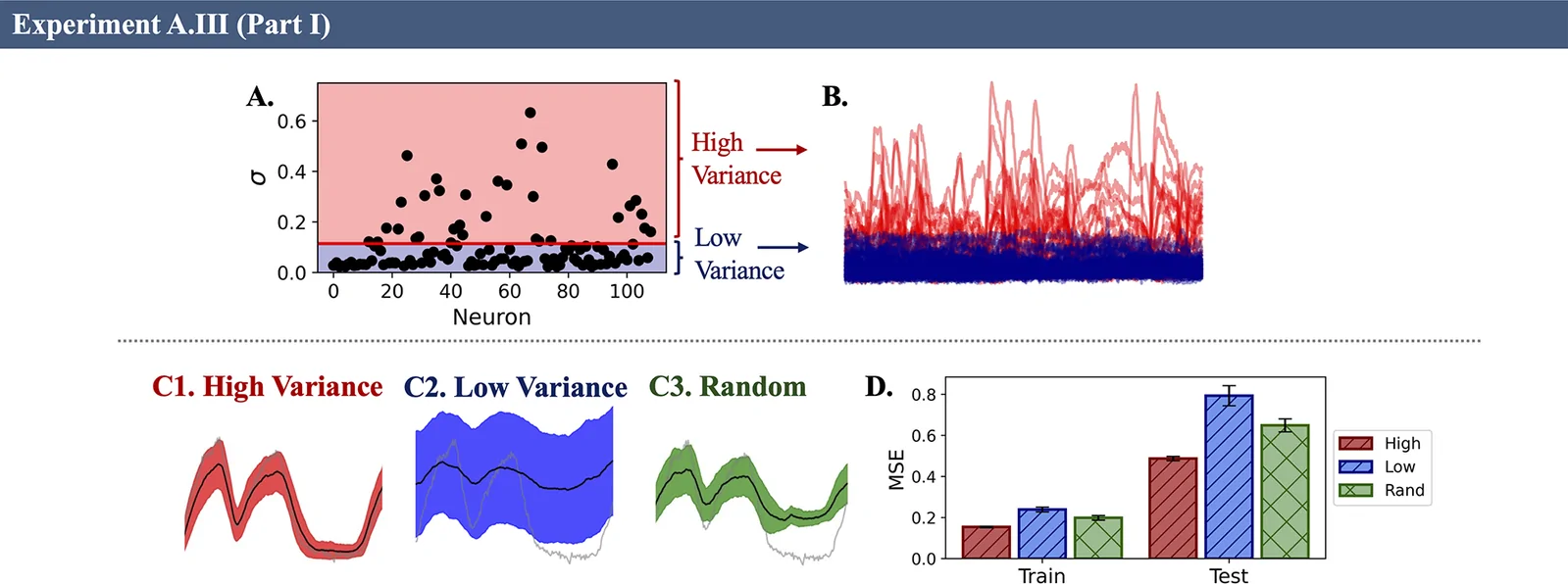
Selecting neurons with highly variable activity improves SHRED performance. (A) Scatterplot of 109 neurons (black dots) showing variability in activity, measured as the standard deviation across 1568 time points. The red line indicates the mean standard deviation. Neurons with values above the average were classified as the HV group and those below were classified as the LV group. (B) Activity traces from HV neurons (red) and LV neurons (blue) illustrating differences in signal variability. (C) A randomly selected neuron trace (grey) and average SHRED reconstruction (black) from 25 trials. Shaded region indicates ±1 s.d. (D) Average train and test m.s.e. for 25 trials using three neurons sampled from the HV, LV or full population (random).

Out of the 109 neurons recorded from W1, approximately half exhibited minimal fluctuations in activity over the entire recording period (figure 4A,B). This suggests that a substantial proportion of neurons remained inactive throughout the trial; thus, the selection of these neurons for population-wide predictions with SHRED may result in sub-optimal results. Standard deviations across time for each of the neurons were calculated, and neurons with activity levels above the mean were classified in the HV group and those below were classified in the LV group. To test the effect of neuronal variability on SHRED performance, three neurons were randomly selected from each of the following groups: HV, LV and the full population (rand). SHRED was trained to reconstruct the entire population activity. We repeated these trials for 25 independent runs, and the corresponding train and test m.s.e. were calculated.

Random selection from the HV group yielded the best overall performance. Across 25 independent runs, the averaged prediction for the HV condition (train: 0.159±0.004 and test: 0.504±0.010) most closely matched the ground truth and exhibited lower variability in performance compared to the LV (train: 0.241±0.012 and test: 0.799±0.049) and rand conditions (train: 0.202±0.011 and test: 0.657±0.032) as shown in figure 4C,D. While randomly selecting from the entire population resulted in lower overall m.s.e. compared to the LV condition, there is still a substantial variability in accuracy across runs. Variance preprocessing is not a prerequisite for SHRED [12], but its utility depends on the dataset. Unlike other applications, primarily in physics, neural activity can often be represented using only a small subset of neurons or channels. Population coding allows complex and dynamic tasks to be captured by the activity of a fraction of neurons. Since SHRED relies on inputs that are dynamically coupled with other recordings, performance naturally declines when inactive neurons or channels are included. Thus, we recommend testing both variance-based selection and random sampling to assess potential performance differences.

Previous applications of SHRED—such as in sea surface temperature, turbulent fluid flow and magnetoencephalography recordings—have demonstrated that as few as three sensors are sufficient to reconstruct whole-system dynamics [43]. To support these results, we then tested the effect of the number of neurons selected on the reconstruction of this immobilized *C. elegans* neural dataset. A total of 15 independent runs were conducted for trials selecting from one, up to five neurons from the HV group. Train and test m.s.e. were calculated and averaged across trials.

**Figure 5.**
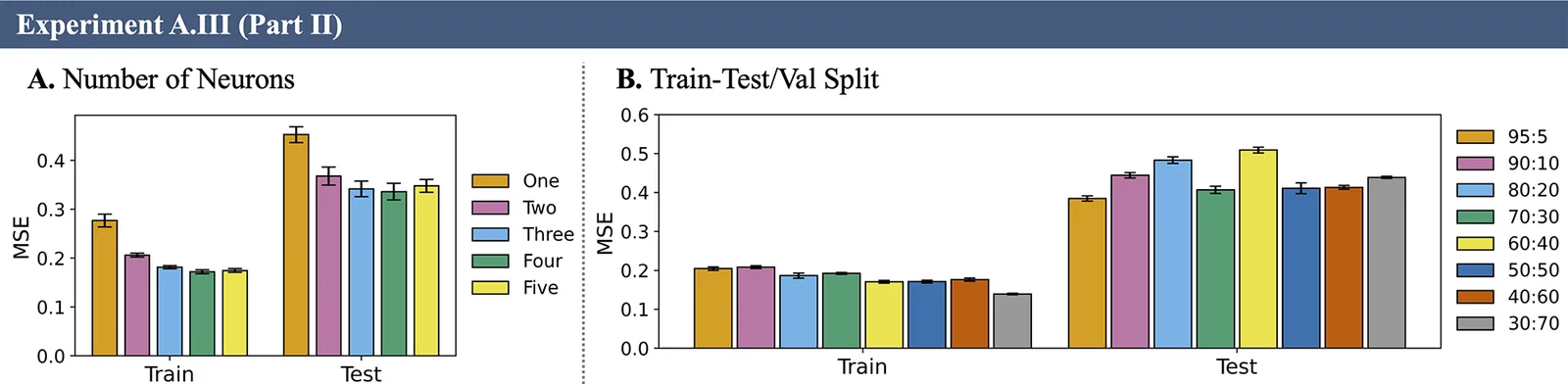
Number of neurons and data split influence SHRED performance. (A) Train and test m.s.e. decrease with neuron count but plateau after three neurons. (B) The data split primarily affects reconstruction accuracy on the test dataset, with minimal impact on the training. A training and test + validation split of 80 : 10 : 10 resulted in the best performance. All trials conducted with variance preprocessing.

As expected, three neurons are sufficient for accurate reconstruction of the entire population dynamics using SHRED. As shown in figure 5, the prediction error plateaus after around three neurons. There is no substantial additional benefit when including more than three neurons. Analogous to how three sensors are sufficient to localize objects in two dimensions, reconstruction of spatio-temporal data also seems to improve with the inclusion of three sensors.

We also tested the effect of data splits on SHRED accuracy. For convention, an x:y split in data represents a split with x% train, y/2% validation and y/2% test. We conducted five independent runs each for the following splits {95 : 5, 90 : 10, 85 : 15, 80 : 20, 70 : 30, 60 : 40, 50 : 50, 40 : 60, 30 : 70} and averaged the train and test m.s.e. Based on the results discussed above, we selected three HV neurons for this analysis. The data split does not seem to have a significant impact on the accuracy of the reconstruction of the training or testing data. Since an 80:10:10 split is customary, we selected this split for the majority of the experiments conducted for the nematode and the mice.

Applications of SHRED on the nematode *C. elegans* dataset demonstrated the flexibility and adaptability of this neural-network architecture. From only three neurons, SHRED could predict population-wide activity for individual worms and across organisms. We next increased the challenge by leveraging SHRED for a more complex dataset: mouse unit and LFP recordings.

### (b) Mouse

The mouse is a popular model organism to study behaviour and human diseases [44]. In recent years, we have seen a rapid rise in techniques for large-scale neural recordings during animal behaviour, opening the doors to understanding network-level neural codes in the mouse brain [45,46]. The next dataset we used is from The Visual Coding—Neuropixels project developed by the Allen Brain Observatory (http://allensdk.readthedocs.io/en/latest/visual_coding_neuropixels.html). The main objective of the Neuropixel project was to study neuronal response to visual stimuli. Mice were presented with various visual cues such as gabors, flashing lights and drifting gratings (DGs). The neural activity—both individual neurons and (LFPs)—in response to these stimuli was recorded from six neuropixel probes inserted throughout the visual cortex.

Each probe contained 374 or 383 channels with each channel containing two bands. The ‘spike band’ measured action potentials of nearby neurons and was recorded at 30 kHz. The ‘LFP band’ measured voltage fluctuations and was recorded at 2.5 kHz. LFPs capture lower frequency voltage fluctuations resulting from transmembrane currents from multiple neurons. Further processing and filtering were conducted to produce the publicly available Neurodata Without Borders files. Pupil diameter measurements were also provided for the duration of the recordings. For convention, individual neurons were referred to as ‘units’ in the spiking dataset since it cannot be guaranteed that each recording originates from a single cell. Therefore, for the following sections, we also refer to neurons as ‘units’.

To simplify analysis, we only selected data from a single mouse. A 96-day-old male mouse (ID 756029989) was selected owing to the quality of its recordings. Both unit and LFP data were used in the following sections to demonstrate the robustness of SHRED. From reconstructing LFP and average spiking data (§3b(i), 3b(ii)) to predicting LFP from either an adjacent neuron's firing rate (§3b(iii)) or a pupil area (§3b(iv)), we applied SHRED in a variety of contexts and saw promising results.

#### (i) SHRED reconstruction of neural response to visual stimuli

**Figure 6.**
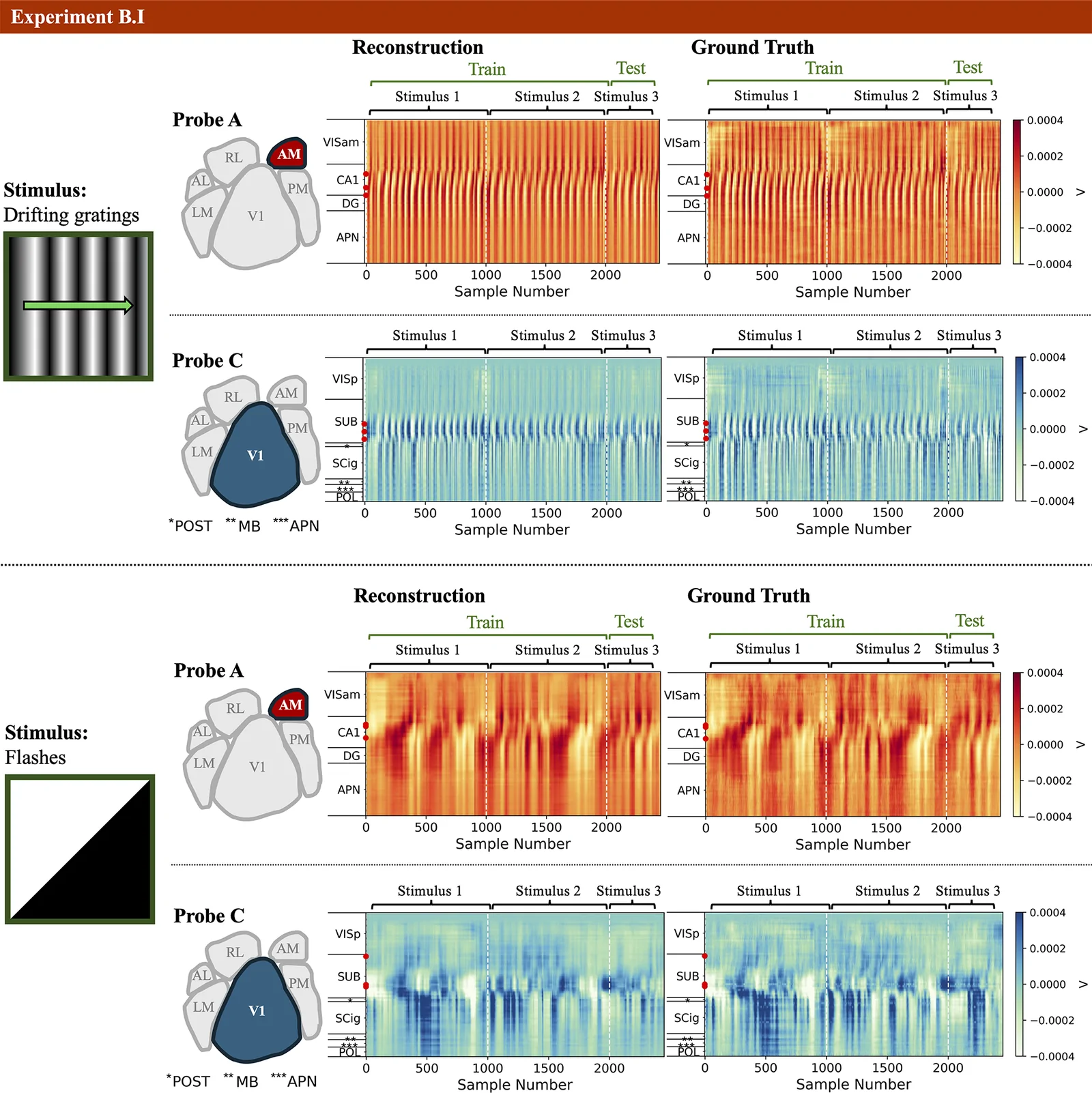
SHRED can accurately reconstruct local field potentail (LFP) in response to a 2-s drifting grating (DG) and a 25-ms light flashing stimulus. Stimulus: LFP recordings were obtained in response to the first three 2-s presentations of a $180^{\circ }$, 0.8-contrast DG stimulus (top) and 25-ms presentations of a 0.8-contrast light flash stimulus (bottom). Probe A: LFPs were recorded from the anteromedial (AM) region of the mouse visual cortex. Red dots on the* y*-axis indicate the three neurons selected from the cornu ammonis 1 (CA1) region for training. Probe C: LFPs were recorded from the primary visual cortex (V1) of the mouse. Red dots on the *y*-axis indicate the three neurons selected from the HV subiculum (SUB) region for training. Reconstruction: Recordings from the first two presentations of the stimulus were used for training, while the third presentation served as the test dataset. For both probes, SHRED accurately reconstructed visually evoked LFP responses. Refer to table 1 Experiment B.I for error metrics. *, postsubiculum (POST); **, midbrain (MB); ***, anterior pretectal nucleus (APN).

Our first objective was to reconstruct LFP responses to visual stimuli. In this section, we obtained data for the first three presentations of (i) a 2-s $180^{\circ }$ DG stimulus and (ii) a 25-ms light flashing stimulus. Visualizations of these stimuli can be found in figure 6. We selected LFP recordings from both probe A and probe C. Probe A was inserted into the anteromedial (AM) region of the visual cortex and contained a total of 74 channel recordings from the AM area (VISam), cornu ammonis 1 (CA1), dentate gyrus (DG) and the anterior pretectal nucleus (APN). Probe C was inserted into the primary V1 and contained a total of 76 channel recordings from the primary visual cortex (VISp), subiculum (SUB), postsubiculum (POST), superior colliculus (SCig), midbrain (MB), APN and the posterior limiting nucleus of the thalamus (POL).

We reconstructed LFP responses recorded on probes A and C during both the DG and flash stimuli. To clarify the set-up, we trained four separate models: (i) probe A LFP with DG stimulus, (ii) probe C LFP with DG stimulus, (iii) probe A LFP with flash stimulus, and (iv) probe C LFP with flash stimulus. For all experiments, the data were downsampled so that each response period contained 1000 time points. Consequently, the 2-s DG response was sampled at 0.5 kHz, whereas the 25-ms flash response was sampled at 4 kHz. The training data were split to include only the first two stimulus presentations. The first half of the third stimulus presentation was set aside as the test dataset, and the latter half of the third stimulus presentation was assigned to the validation set. For reconstructions of probe A, we randomly selected three channels from the CA1 region of the hippocampus and trained with a lag of 100 for 200 epochs. For probe C, we randomly selected three channels from the SUB and trained with a lag of 100 for 200 epochs. No variance preprocessing was conducted for either stimuli.

SHRED more accurately reconstructed LFP data from probe A compared to probe C for both stimulus types. In particular, DG LFP recorded on probe A resulted in the most accurate reconstruction (train: 0.355; test: 0.391) as shown in table 1. By looking at the original dataset, it is clear that there is a simple oscillatory pattern in the voltage which is a fairly straightforward task for many sparse-sensing algorithms. However, the other reconstruction tasks were more difficult, especially the LFP recorded from probe C. Some sensors in the VISp and SUB exhibited a heightened level of activity which SHRED was unable to reconstruct. Nevertheless, general patterns in activity are conserved in the reconstruction of both probe A and probe C activity for all stimulus types. The similarity between train and test m.s.e. further highlights SHRED's generalizability (table 1B.I). This algorithm can learn larger scale spatio-temporal patterns in population codes. By leveraging these patterns, SHRED can reconstruct whole-population activity from only three sensors.

These preliminary results suggest that SHRED could help reduce the impact of invasive procedures. Once trained, the model can predict brain activity in regions not directly recorded, potentially allowing for fewer sensors and thereby decreasing the invasiveness of future experiments.

**Figure 7.**
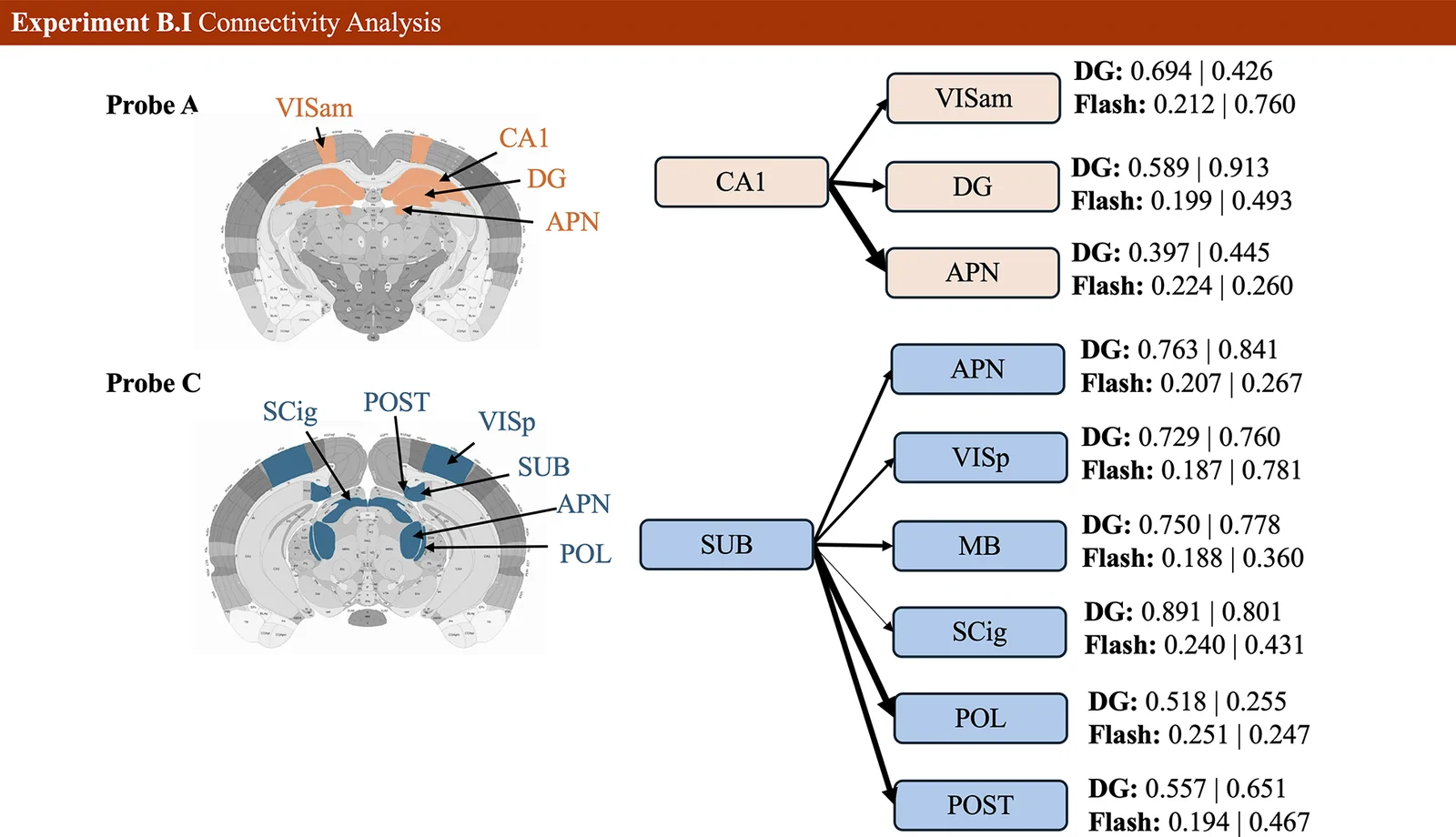
Reconstruction accuracy may correlate with functional connectivity or anatomical proximity. Left: Diagram of the regions recorded by probe A and probe C. Right: Connectivity diagram showing links from the CA1 and the SUB to other brain regions. Arrow widths are scaled according to hypothesized connectivity. Train | test m.s.e. values are shown for both the DG and flash stimulus conditions. Note that DG in the orange box refers to the dentate gyrus.

In addition to aiding in non-invasive or minimally invasive experimental procedures, SHRED may provide some insight into anatomical connectivity through its reconstruction accuracy. To explore this potential application, the reconstruction accuracy was calculated for each region separately as shown in figure 7. To limit the influence of the sample number on the error metric, only eight sensors from each region in probe A and two sensors for each region recorded by probe C were used to calculate the relative error. From there, an estimate of the connectivity strength was calculated by taking a weighted sum of the train and test m.s.e. as shown below:

$$E=0.8*\left({\text{m.s.e.}}_{{\text{train}}_{\text{dg}}}+{\text{m.s.e.}}_{{\text{train}}_{\text{flash}}}\right)+0.2*\left({\text{m.s.e.}}_{{\text{test}}_{\text{dg}}}+{\text{m.s.e.}}_{{\text{test}}_{\text{flash}}}\right).$$

A proxy measure for connectivity was defined as such:

C=1/E.

The thickness of the arrows in figure 7 is scaled according to the hypothesized connectivity (table 2). These results suggest that dynamics in the CA1 are most closely coupled to the APN and the dentate gyrus (DG). While no evidence of strong CA1–APN afferents are reported in the current literature, several studies highlight the role of the APN in sensory processing—particularly somatosensation—with projections into higher-order thalamic relays [47,48]. By contrast, evidence of CA1–DG connections is more concrete. Although not direct, the hippocampal trisynaptic circuit (DG → CA3 → CA1) is well-established and studied in the context of sensory-related learning [49,50]. Afferants from DG to CA1 may also influence dynamic coupling between these regions, resulting in more accurate reconstructions. Interestingly, reconstruction accuracy in this case appears to align more closely with anatomical proximity than with established functional connectivity. The CA1, DG and APN are all subcortical regions without separation by white matter, in contrast to VISam. Further analysis will be required to validate these observations.

**Table 2.** Hypothesized connectivity values for probes A and C.

CA1 → VISam	CA1 → DG	CA1 → APN			
1.040	1.097	1.568			
SUB → APN	SUB → VISp	SUB → MB	SUB → SCig	SUB → POL	SUB → POST
1.002	0.961	1.022	0.869	1.397	1.213

For probe C, the connectivity calculated from the reconstruction accuracy suggests that the SUB is most closely linked to the POST and the POL. The POST, one of the four regions of the subicular cortex, receives projections from the hippocampal formation, the cingulate cortex and the thalamus [51]. Because the SUB and POST are in close proximity (figure 2), some signal leakage across probes may contribute to the observed coupling. Importantly, the reconstruction of the POL was the most accurate. While numerous studies support subiculum–thalamus connectivity with the anterior thalamus [52–54], these results raise the possibility of a broader interaction between these regions. Overall, reconstruction accuracy does not map cleanly onto known anatomical connectivity, but the error metrics may nonetheless provide insight into functional coupling not yet fully characterized.

#### (ii) SHRED reconstruction of average unit responses to visual stimuli

We next aimed to reconstruct averaged unit spike trains in response to all 2-s DG and 25-ms flash stimuli. Spike train data are inherently stochastic and consist of discrete, binary events, presenting a significant challenge for many machine learning methods. We averaged spike train data across all presentations of each stimulus type to obtain a more continuous estimate of each unit's response. We obtained recordings from units with signal to noise ratios greater than 3. This cut-off was recommended in the documentation of the dataset. For each 2-s presentation of the DG stimulus and 25-ms flash stimulus, we obtained 1000 time points of spiking data for 243 units. We then averaged this data across presentations to calculate the average unit responses. Note that the sampling rate (SR) differs between the stimuli. Reconstruction of the flash response with the same SR resulted in subpar results owing to limitations in data. Therefore, the SR for the flash stimulus was increased.

The data were preprocessed and split into 102 HV units and 141 LV units. Three units with high variability in activity were randomly selected from the HV group, and the model was trained for 200 epochs.

**Figure 8.**
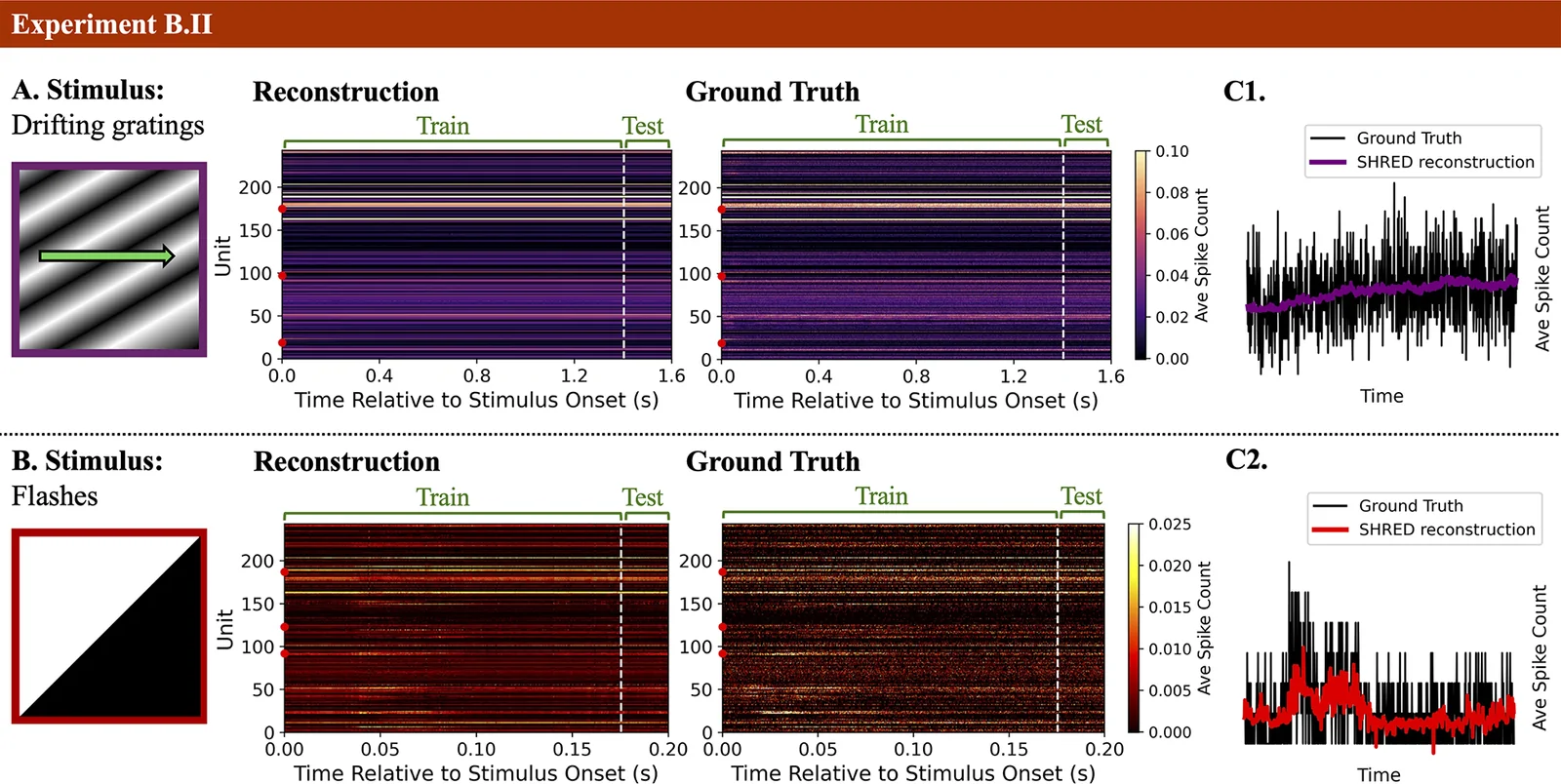
SHRED accurately reconstructs and filters high-frequency recordings of averaged unit activity. (A) SHRED reconstruction of average unit activity for all 2-s trials with DG stimuli. (B) SHRED reconstruction of average unit activity for all 25-ms trials with light flashing stimuli. (C1,C2) Average activity (black) and SHRED reconstruction (purple/red) of a randomly selected neuron. For both stimuli, averaged unit recordings contain high-frequency fluctuations which are filtered out in the SHRED reconstruction. Overall trends in activity are well-preserved. Red dots on the *y*-axis indicate sensors selected as input. Refer to table 1 Experiment B.II for error metrics.

SHRED more accurately reconstructed the average unit response to the drifting gradient stimulus compared to the flashes. This is most likely owing to lower fluctuations in individual spiking activity across time for the DG stimulus compared to the flash (figure 8). We observed lower fluctuations most likely owing to two main reasons: (i) the DG unit response is sampled with a lower SR and (ii) responses to DGs of different orientations, speed and contrasts are averaged together, smoothing out the unit's response. For example, consider a unit with a receptive field sensitive to light located in the bottom left-hand corner of the screen. When gratings are presented at different orientation and at different speeds, it is plausible that the receptive field will always be lit in at least one of these presentations. Therefore, averaging the data may result in a smoother activity trace, setting up an easier reconstruction task for SHRED.

While the reconstruction errors are higher for the flash stimulus (table 1B.II) the example traces of unit 150 in figure 8C1,C2 show that general activity patterns are preserved. While individual spikes are not reconstructed, SHRED learns the time course of when the unit is firing more frequently. From this result, we hypothesized that the model is learning the overall firing rates rather than the timing of the individual spikes. Therefore, in the following section, we focus on reconstruction of LFP from firing rate.

#### (iii) SHRED reconstruction of local field potential from individual neuron firing rate

In §3b(ii), we observed that SHRED learns overall firing rates of individual units when reconstructing averaged spike data. Therefore, we then hypothesized that SHRED could reconstruct LFP from solely the firing rate. Although LFP recordings contain substantially higher-frequency fluctuations than the firing rate, our focus was to first reconstruct the lower frequency signals. We obtained a spike train from a single unit from probe A in response to the first 2-s, $180^{\circ }$, 0.8 contrast DG stimulus. This unit was selected because of its high firing rate (greater than 10). We then obtained the LFP recording from the same channel, which reflects voltage fluctuations in the extracellular space near the selected unit.

**Figure 9.**
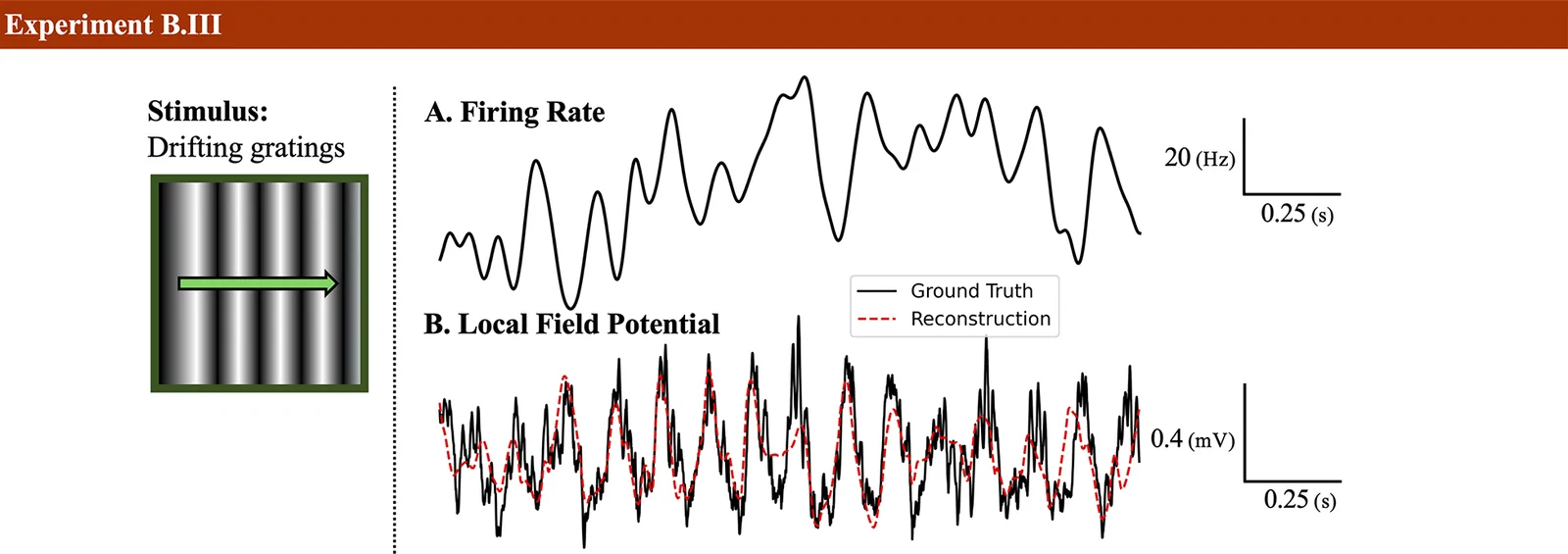
SHRED can reconstruct LFP from firing rate of nearby neuron. (A) Firing rate of a neuron in the visual cortex located near the probe inserted in the AM region served as the input to the SHRED model. (B) The SHRED model was trained to reconstruct LFP recording closest to the neuron selected as input. The model reconstruction (red) closely follows the general patterns of the LFP recoding (black). Red dots on the *y*-axis indicate sensors selected as input. Refer to table 1 Experiment B.III for error metrics.

The firing rate of the selected unit was calculated with a Gaussian filter. Firing rate (v) of a neuron is defined as

$$v=\frac{n_{s}}{t},$$

where $n_{s}$ is the number of spikes in time t. To obtain an accurate measurement of the spike rate, we used a one-dimensional Gaussian filter. The SR (sr) for the unit data was down sampled to around 1.25 kHz. The standard deviation for the Gaussian was set to

$$\sigma =\frac{0.02}{\text{sr}},$$

thus the spike train was smoothed over a 20-ms window. After applying the filter, we multiplied the filtered trace by the SR to obtain the firing rate in spikes s^–1^. The smoothed firing rate is shown in figure 9A. We included 2002 time points in the training, 250 in the validation and 250 in the testing dataset. SHRED was trained to predict the LFP and reconstruct the firing rate.

From just the neuron firing rate, SHRED can predict LFP (table 1B.III). The SHRED reconstruction of the entire dataset shown in figure 9 closely follows the data. While there are some deviations between the predicted and the true LFP, overall activity trends are well-preserved. It is evident that the firing rate is dynamically coupled to the LFP, making this a suitable setting for applying SHRED. Based on the conclusions of this experiment, we hypothesized that any dataset that is dynamically coupled to neural recordings should be sufficient to reconstruct population-wide neural activity, even if it is simply a proxy of neural activity. Therefore, in the next experiment outlined in §3b(iv), we reconstructed LFP data from just the pupil area recording.

#### (iv) SHRED reconstruction of local field potential from pupil area response to a visual stimulus

Previous experiments demonstrated SHRED's ability to reconstruct neural activity from inputs directly correlated with neural activity. Here, we extend this approach by attempting to reconstruct LFP recordings from the time series of the pupil area. We hypothesized that pupil size reflects central nervous system activity, consistent with the proposed model in Raut *et al.* [55]. Because LFPs represent neural responses evoked by visual stimuli that are first encoded through the retina and gated by the light passing through the pupil, we proposed that the pupil area should be dynamically coupled to the evoked neural response. Therefore, in this experiment, we aimed to reconstruct LFP recordings from just the pupil area measurements. Probe A LFP and pupil area recordings were obtained from the first presentation of the 2-s, $180^{\circ }$, 0.8 contrast DG stimulus. To account for the different SRs, we applied a cubic spline to the pupil area measurements. The pupil area recordings were upsampled, to test whether finer-scale trends in the LFP data could be reconstructed. Thus, pupil recordings were obtained for the same time points as the LFP recordings. We split the data into 75% train, 12.5% test and 12.5% validation. With a lag of 50, pupil area recordings served as the input to the model which was trained to predict the LFP and reconstruct the pupil area recordings.

**Figure 10.**
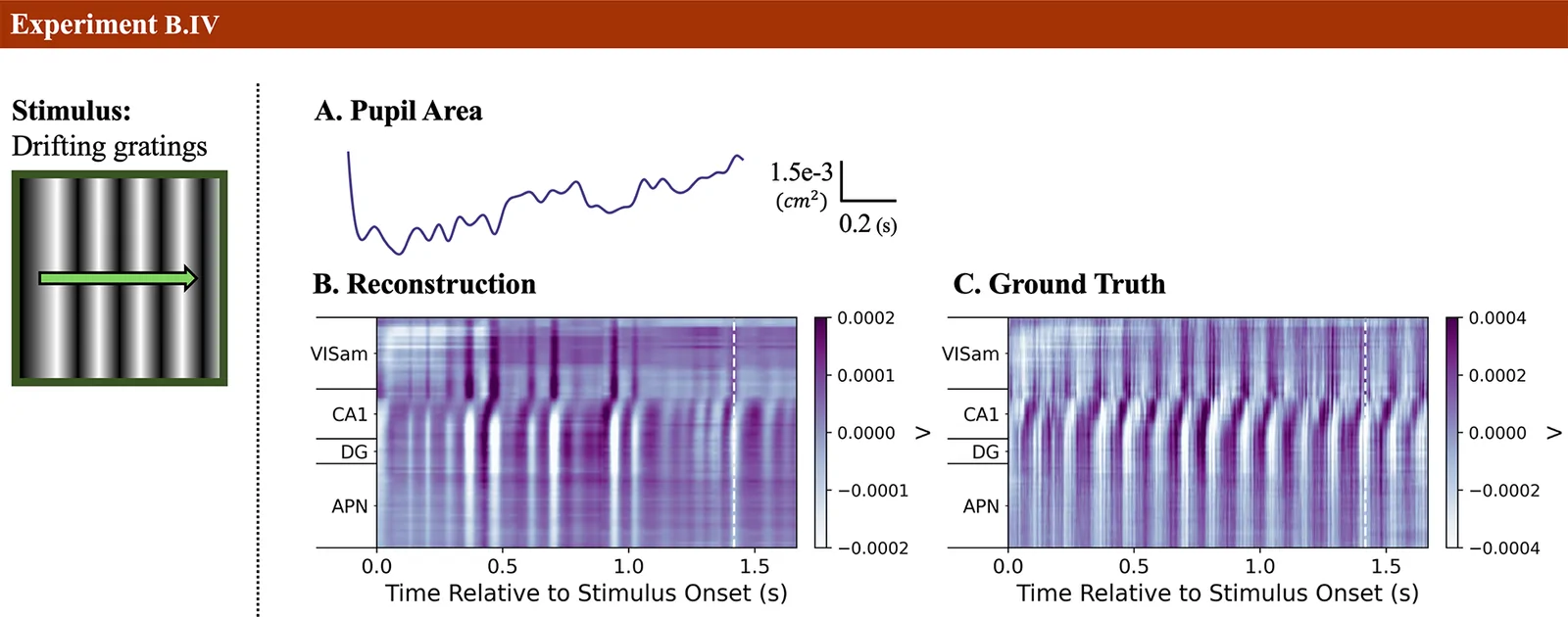
SHRED has potential to reconstruct LFP activity from pupil area. (A) Pupil area in response to a 2-s DG stimulus served as the input to the SHRED model. (B) The SHRED model was trained to reconstruct LFP activity from a probe inserted in the AM region of the mouse visual cortex. The reconstruction successfully captures coarse fluctuations in spatial and temporal activity. Red dots on the *y*-axis indicate sensors selected as input. Refer to table 1 Experiment B.IV for error metrics.

The reconstruction of population dynamics from pupil area introduces additional challenges compared to the tasks outlined in the previous section. Pupil area is not a direct correlate of neural activity. However, SHRED performed surprisingly well. The reconstruction shown in figure 10 deviates from the ground truth, but the peaks and troughs are still maintained. Train and test m.s.e. are presented in table 1B.IV. While there is still more work needed to improve the prediction accuracy, this baseline result highlights the versatility and robustness of the SHRED architecture.

Applications of SHRED to the mouse dataset highlighted its diverse usage. Not only is SHRED capable of reconstructing population neural activity from a few sensors, it is also capable of reconstructing population dynamics from any coupled measurements. While cross-individual predictions were not feasible owing to limitations of the dataset, individual reconstructions still offer significant value. They can help reduce the need for invasive recording procedures and even enable recovery of data from faulty or missing sensors. A model trained to predict full population activity from recordings in a single brain region, or even from proxy measures such as pupil area, which are easy to acquire, opens the door to a wide range of applications in neuroscience and clinical settings. By learning the patterns and connections in these datasets, SHRED was capable of predicting general brain activity from unit firing rate and pupil area measurements. To further explore the versatility of SHRED, we next reconstructed a video of the zebrafish forebrain activity.

### (c) Zebrafish

The zebrafish, a small freshwater species in the minnow family, is a widely used model organism for studying vertebrate development and, more recently, systems neuroscience [56,57]. Zebrafish offer several advantages in research, one of which is their transparency during early developmental stages, enabling clear and accessible imaging [56,58]. Recent studies have focused on understanding neuronal ensembles code for sensorimotor pathways [5,59–62]. With improvements in imaging technologies, high-speed light-sheet microscopy can now be implemented to simultaneously record from almost all neurons in the zebrafish. This large-scale recording allows for complex analysis of activity patterns in the whole brain [63].

For our third model system, we looked at a video of forebrain activity of a zebra fish obtained using high-speed light-sheet microscopy. This video was made publicly available by Ahrens *et al.* [63]. Neural projections were genetically encoded using a calcium indicator GCaMP5G. The 1041.859-s video contained 750 frames indicating a frame rate of approximately 0.72 Hz. We then converted the video to greyscale and cropped each frame to include 472×600 pixels, thus a total of 283 200 pixels. The original video (forebrain.avi) was cropped to only include the left-hand side which shows the dorsal maximum-intensity projections of whole-brain functional activity and maximum-intensity projections of the reference anatomy (grey).

Variance preprocessing was applied to this dataset to ensure that the background of the forebrain slice was not included as the input. There were 92 634 pixels in the HV group and 190 566 pixels in the LV group. We aimed to reconstruct this video from the time series of just 250 pixels. More sensors were needed as input compared to the previous sections owing to the greater number of total pixels. However, even with this increase, we still only included approximately 0.9 % of the total sensors as input. We split the data to include 700 time points in the training set, 25 time points in the testing set and 25 time points in the validation set and trained the model for 200 epochs.

**Figure 11.**
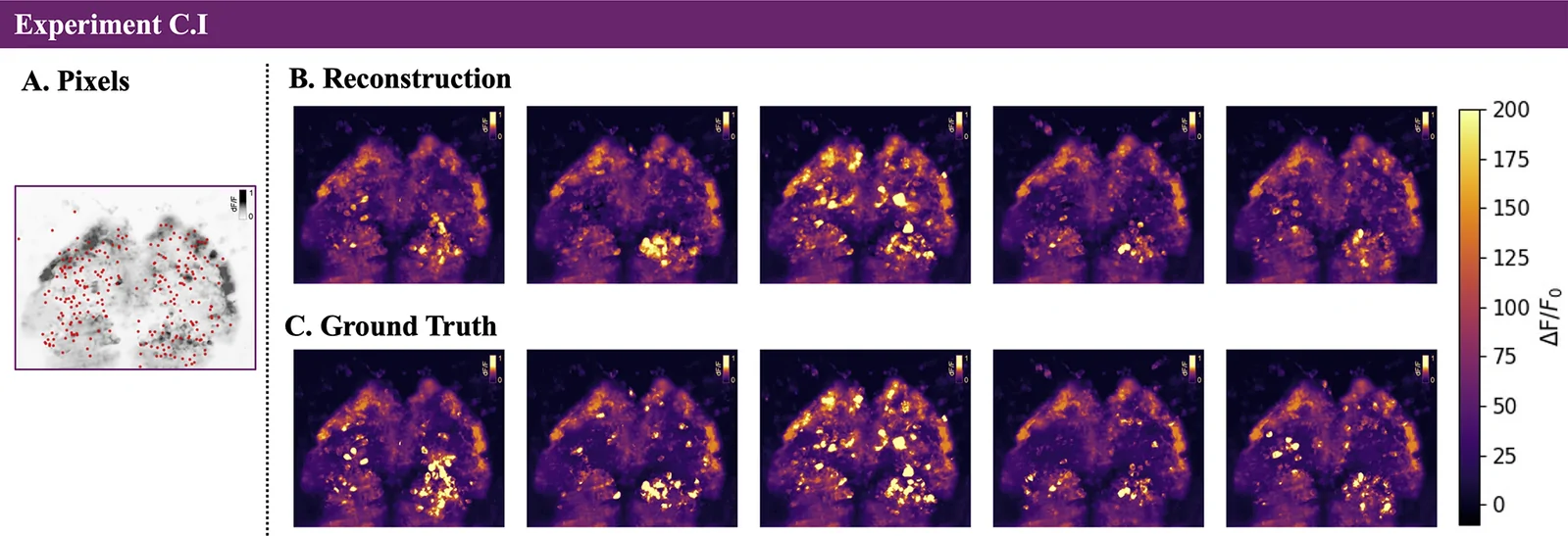
SHRED accurately reconstructed a video of zebrafish forebrain activity. (A) Pixels selected as input for the model. (B,C) SHRED reconstruction of six time points (top) follows general trends in activity of the original (bottom). Refer to table 1 Experiment C.I for error metrics.

SHRED struggles to reconstruct individual changes in pixels, but captures flares of activity with great accuracy (figure 11). Train and test m.s.e. are presented in table 1C.I. The overall shape of the forebrain was preserved, and regions with increased activity were reflected in the reconstruction. In this section, we showed that SHRED can be used to reconstruct videos. SHRED is versatile and can be applied to a variety of complex datasets with minimal hyperparameter tuning.

### (d) Control comparison

We also performed linear regression as a baseline for each experiment, using the same neurons or sensors provided to SHRED as input. SHRED outperformed this baseline in nearly all tasks based on m.s.e. However, m.s.e. alone was insufficient to capture the differences and strengths of each model. In this section, we therefore evaluate performance using the power spectral density (PSD), which highlights the reconstructed frequencies relevant to the dataset. It should be noted that neural networks generally act as bandpass filters owing to the spectral bias in learning when using m.s.e. in training [64]. Regardless, SHRED generally does a better job of preserving the frequency components and the associated PSD than simple linear models.

**Figure 12.**
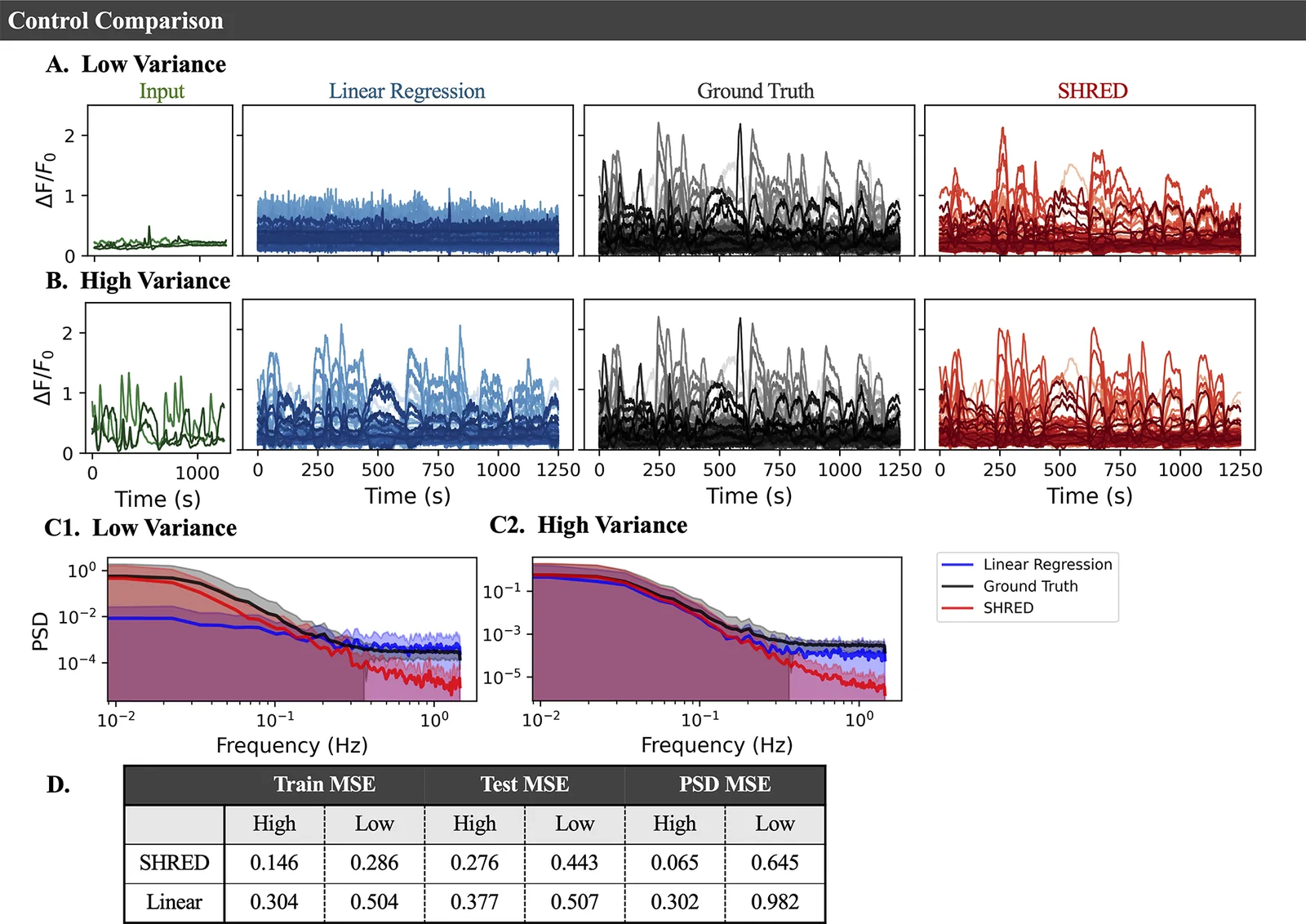
SHRED outperforms a linear regression baseline. (A,B) Reconstructions from three neurons selected from either the (A) LV or (B) HV groups, showing ground truth activity alongside linear regression and SHRED outputs. (C) Power spectral densities (PSDs) of SHRED (red) and linear regression (blue) reconstructions compared to the ground truth (grey). (D) Summary of error metrics.

As shown in figure 12, SHRED outperformed linear regression in both training and test reconstructions of the first *C. elegans* worm from experiment A.I (§3a(i)). These results were obtained using variance preprocessing with three neurons selected from the HV group. With the same neurons, linear regression performed surprisingly well (figure 12B,C2,D). Nonetheless, further analysis indicates that SHRED (i) is more robust to neuron selection and (ii) reconstructs lower-frequency patterns more accurately.

We compared SHRED and linear regression under two experimental conditions: training on three neurons from either the LV or HV subsets. With LV input, the linear model fails to reconstruct the full state space, reflecting its inability to extrapolate beyond the given signals. By contrast, SHRED accurately reconstructs population activity, including HV neurons (figure 12A,C1). PSD analysis further highlights this difference: the linear model struggles to capture lower-frequency components, whereas SHRED closely follows the ground truth, even with limited input. Both the PSD and individual traces also show that SHRED acts as a filter, suppressing high-frequency fluctuations that persist in the linear model.

When HV neurons are used as input, the performance of both models improves (figure 12B,C2). However, the linear model benefits more from this optimal selection, underscoring SHRED's robustness to neuron choice. The PSD in C2 indicates that both models capture low-frequency patterns more accurately, yet SHRED still outperforms linear regression in both train/test m.s.e. and PSD m.s.e. (figure 12D). This advantage arises because SHRED reconstructs lower-frequency dynamics more precisely and on the correct timescale (note the log scale in figure 12C1 and C2).

For this dataset, the primary goal was to recover the general activity patterns and timing of neuronal activation. High-frequency fluctuations are likely noise and therefore less critical to reconstruct. If both low- and high-frequency components were desired, a potential future direction would be to combine the strengths of the two models. For example, SHRED could be used for low-frequency dynamics and linear regression for high-frequency detail. Nevertheless, for the purposes of these experiments, SHRED consistently outperforms the linear regression baseline, even without optimal sensor input.

When applying these models in a biological context, robustness to sensor selection is critical. If future applications aim to support minimally invasive or non-invasive sensing, the objective is to reconstruct activity from sensors that are easy to access and record from, not necessarily the sensors with the highest quality recordings.

**Figure 13.**
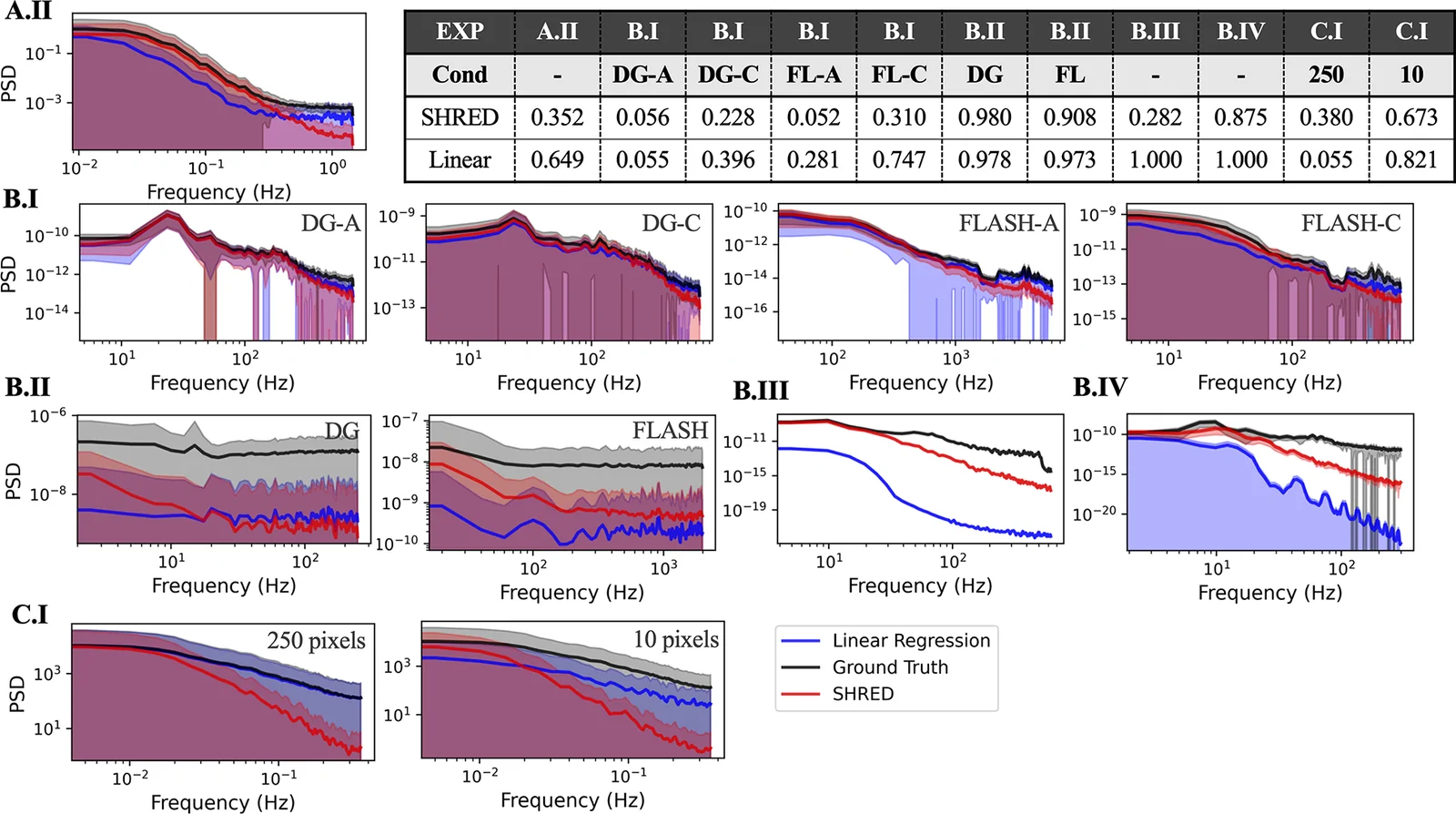
Baseline comparison for experiments in §3a–c. PSDs of SHRED (red) and linear regression (blue) reconstructions compared to the ground truth (grey) for each experiment. Error metrics are presented in the table at the top. DG, drifting grating.

A linear regression baseline was also applied to the experiments in §3a,b, with PSDs and errors shown in figure 13. SHRED outperforms the linear model in all experiments except B.I DG-A, B.II DG and C.I-250 pixels. Experiment B.I DG-A corresponds to reconstruction of mouse LFP recorded on probe A during the DG stimulus (figure 6), which exhibits a strong oscillatory pattern readily captured by linear transformations from the input time series. Similarly, experiment B.II DG explores the average unit reconstruction during the DG stimulus (figure 8), where units display a sustained level of activity that can be well approximated by a line. These cases therefore favour linear regression, as reflected in the error metrics. By contrast, when more challenging datasets are considered for the same tasks (e.g. experiment B.I DG-C and flash-A/C; exp. B.II flash), SHRED outperforms linear regression. For the video reconstructions in experiment C.I, the error of the linear model increases substantially when fewer pixels are used as input. Overall, SHRED demonstrates greater versatility and is less constrained by input quality or quantity. Similar to what was observed in figure 12, lower frequency patterns in data are better reconstructed by SHRED for all experiments presented in this manuscript thus far. More extensive comparisons of SHRED to other models have been conducted by Tomasetto *et al.* and Williams *et al.* [15,43].

### (e) Human locomotion

In the final dataset for consideration, we highlight the recent work of Ebers *et al.* [13] where SHRED was used for mapping temporal sensor measurements to behaviour. To emphasize, these results are reprinted from an already published study [13]. We include this work here to further demonstrate the potential of SHRED for cross-individual transfer. Datasets recorded from the same sensors across different individuals are rare, making this dataset and its analyses particularly valuable for showcasing SHRED's generalizability. Specifically, SHRED is used with mobile sensing in order to monitor human locomotion and biomechanics. The tracking and analysis of human motion are critical for many tasks, including the monitoring of disease progression, guiding rehabilitation treatment, evaluating sports performance and informing assistive device design. Tracking motion in general is an expensive endeavour requiring a myriad of sensors. However, with SHRED one can replace the array of sensors monitoring motion with a single smart watch and its accelerometer. A smart watch is accessible and easy to implement without invasive procedures. The dataset for this mobile SHRED study [13] is an open-source dataset that captures non-disabled human biomechanics during steady, rhythmic walking; such an approach can be generalized to other motion-tracking applications like robotic manipulation or computer animation. We use the time series of joint kinematics from 12 non-disabled adults (six female/six male; age 23.9 ± 1.8 years; height = 1.69 ± 0.10 m; mass = 66.5 ± 11.7 kg) [65]. Participants walked at a self-selected speed (speed = 1.36 ± 0.11 m s^–1^) on a split-belt instrumented treadmill (Bertec Corp., Columbus, USA) for 6 minutes. While kinematics can be captured with multiple modalities, gold-standard marker data were recorded using a 10-camera optical motion capture system. Kinematics were estimated from marker data using the inverse kinematics algorithm in OpenSim 3.3 with a dynamically constrained 19 degree-of-freedom skeletal model scaled to each participant [66,67]. The SHRED work evaluated the 18 kinematic features previously calculated in the open-source dataset, which included three translational degrees of freedom at the pelvis, pelvis tilt/list/rotation, lumbar extension/bending, bilateral hip flexion/adduction/rotation, bilateral knee flexion and bilateral ankle dorsiflexion. Kinematics were low-pass filtered at 6 Hz using a fourth-order Butterworth filter [65]. An important distinction with this human biomechanics data, as opposed to the neural data used earlier, is that marker data recorded during motion capture is in a global coordinate frame, but through the standard procedures for marker data processing are transformed into kinematics in a local coordinate frame relative to the human subject [68].

For the population-based biomechanics models, a reconstruction mapping was trained using 11 of the 12 participants’ kinematic data, for a one-subject hold-out test using each modelling paradigm. Two combinations of dynamic trajectory inputs were tested: (i) three non-randomly chosen kinematic features (right hip flexion angle, right knee flexion angle and right ankle dorsiflexion angle) and (ii) one non-randomly chosen kinematic feature (right ankle dorsiflexion angle).

**Figure 14.**
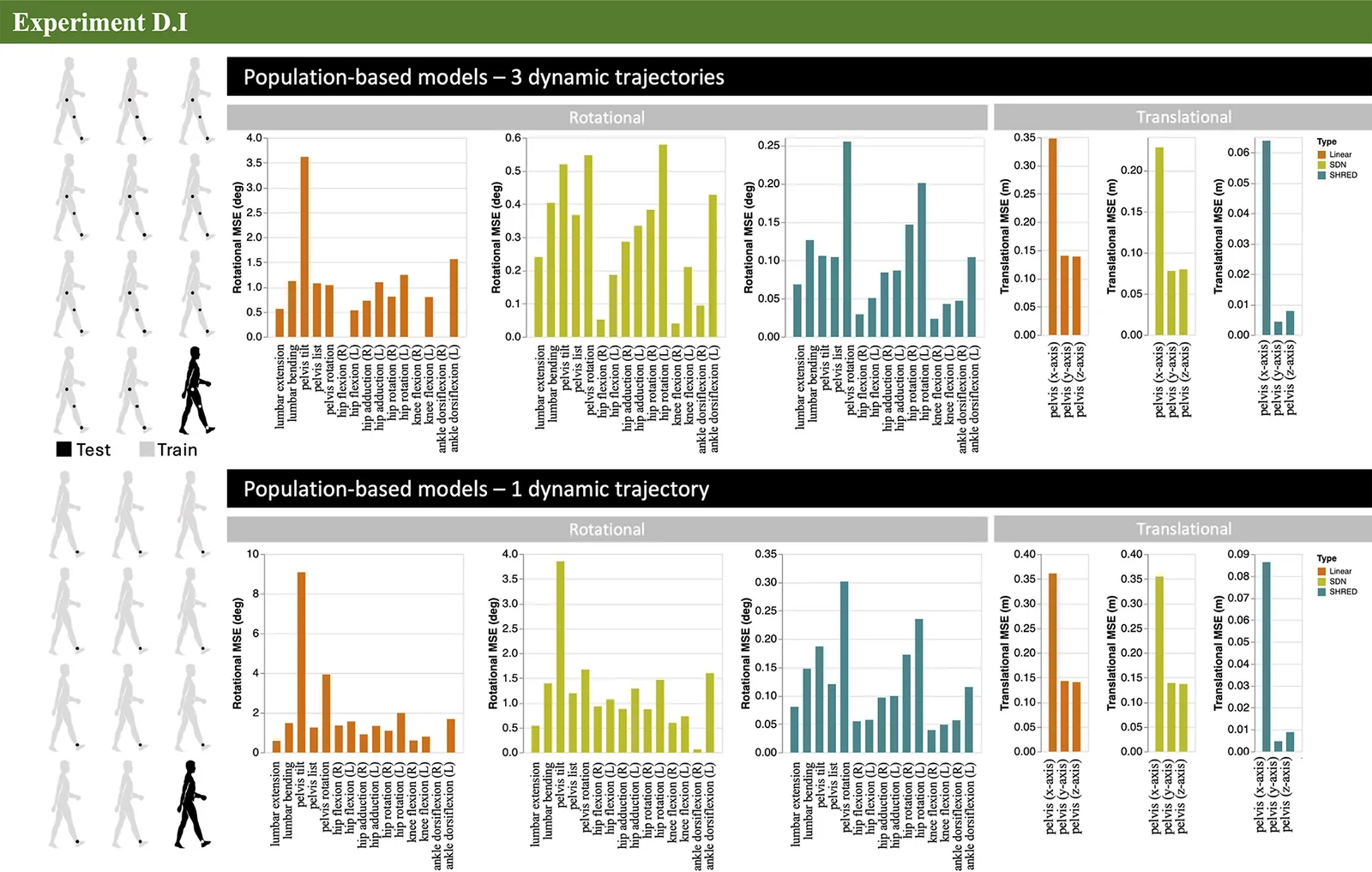
Visualization of population-based results from Ebers *et al.* [13] for reconstructing human biomechanics [65]. The population models were trained using 11 of the 12 individuals’ data and were evaluated by reconstructing the held-out subject's full measurement data from their sparse input sensor(s); performance was calculated by using m.s.e. Rotational and translational kinematic states are displayed separately, as rotational states use units of degrees while translational states use units of metres. Three modelling paradigms were trained to learn the mapping (presented left to right): linear regression in orange, a SDN in green and a shallow recurrent neural network (SHRED) in teal. Note the independent *y*-axes and respective scales for each graph. Top: Three dynamic trajectories were purposefully chosen as sensor inputs to the reconstruction mapping: right hip flexion angle (degrees), right knee flexion angle (degrees) and right ankle dorsiflexion angle (degrees). Bottom: One dynamic trajectory was purposefully chosen as sensor inputs to the reconstruction mapping: right ankle dorsiflexion angle (degrees). (Reprinted with permission from Ebers *et al.* [13].)

Figure 14 summarizes the performance of SHRED relative to the SDN and linear models for reconstructing human motion within a population. SHRED, which consists of both a recurrent neural network and a SDN, far outperforms the SDN and linear model regardless of number or choice of input sensors. Using three mobile sensors, SHRED has errors less than 0.098±0.062 degrees, which was on average 3.3× more accurate than the SDN and 9.6× more accurate than the linear model. Using a single mobile sensor, SHRED has errors less than 0.121±0.073 degrees, which was on average 9.8× more accurate than the SDN and 14.8× more accurate than the linear model. These results demonstrate that SHRED can enable rapid generalization (parametrization of dynamics) for data outside the training set, which may be especially useful in human biomechanics where population-level models can be used to estimate parameters for a new individual (e.g. a new patient visits a clinic for the first time).

## 4. Conclusions and discussions

Modern neuroscience and behavioural sciences are now supplemented with exceptional datasets which measure a diversity of quantities, from individual neurons to whole-brain activity to behavioural outcomes. Such emerging datasets, which are now routinely in the open source domain, offer significant scientific opportunities for learning and discovery in the biological sciences. Specifically, there are modern data-driven mathematical architectures and algorithms capable of extracting interpretable generative models from the diversity of data collected. Moreover, it is ideal if cheap and non-invasive sensing can be used instead of expensive and invasive sensors. The SHRED architecture introduced in this work provides a viable pathway towards using a highly limited number of sensors for reconstructing the full high-dimensional state space of either neural activity or behavioural responses. This is demonstrated on a number of toy models in biology and on behavioural data for locomotion.

SHRED is an exceptionally flexible architecture, being now used for many engineering and physics applications [12–16]. Moreover, it has been demonstrated to have a globally convex landscape which allows for rapid training with little or no hyper parameter tuning [16]. SHRED is ideal for applications in the biological sciences which require flexibility as many applications are in low-data limits with large measurement noise. SHRED can operate in this difficult data limit as easily as in the high-data limit. Importantly, because of the globally convex landscape and ability to train with compressed data [12,15], training SHRED with neural data can be accomplished on laptop-level computing, even for exceptionally large datasets.

SHRED is demonstrated in this work to have the capability of mapping limited neural or behavioural recordings accsurately to full state space measurements. The success of the method allows for deployment in practice to new patients or model organisms. For both the *C. elegans* and biolocomotion examples, the ability of SHRED to be deployed in a parametric fashion affords many opportunities in clinical settings where non-invasive measurements can be used to approximate difficult or invasive measurements. In future work, learning generative models of the dynamics will be explored with the goal of extracting even more meaningful insights into biological activity [16]. Thus, SHRED overall is an emerging deep learning algorithm which is well suited for diverse biological data.

## Figures and Tables

**Figure 15.**
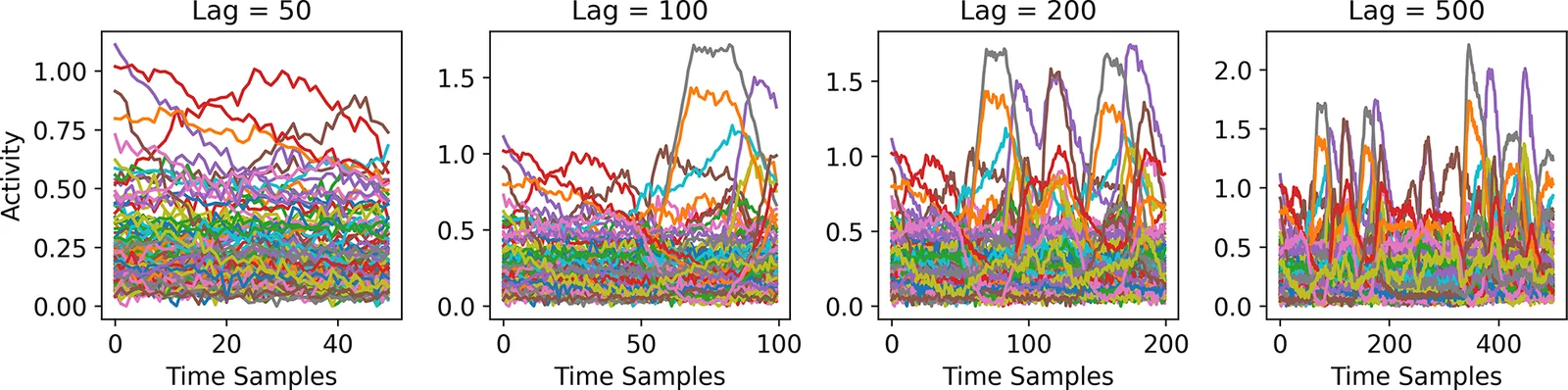
Lag comparison for worm 1.

**Figure 16.**
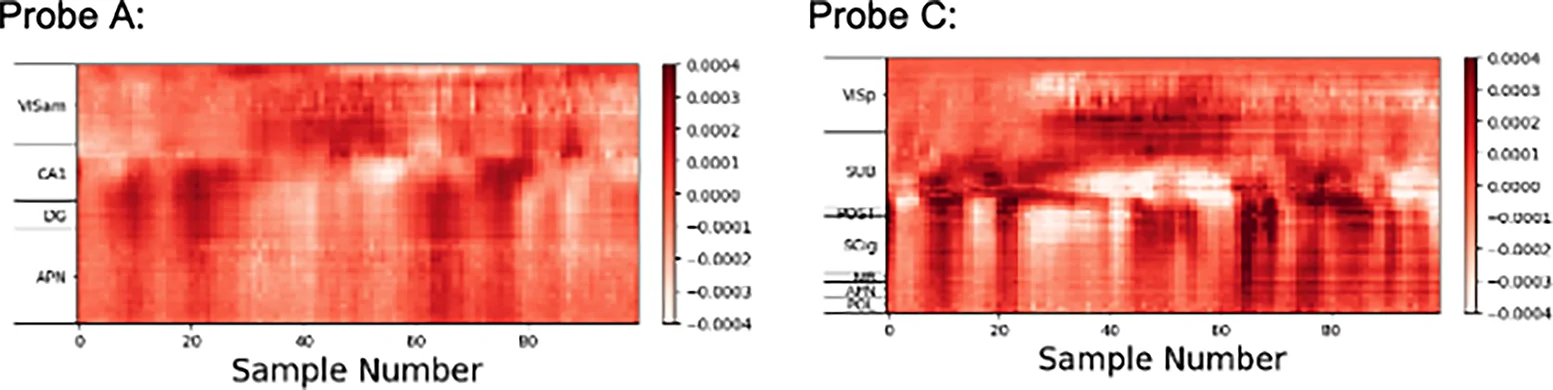
100 time points for probe A and probe C LFP recording to DG stimulus.

## Data Availability

All code and data are publicly available. Figures and experiments can be reproduced from the following GitHub: http://github.com/amysrude/neuralSHRED. The datasets used in the analyses are from the following sites. *C. elegans*: http://osf.io/2395t/. Mouse: download data: http://allensdk.readthedocs.io/en/latest/_static/examples/nb/ecephys_data_access.html. Documentation: http://allensdk.readthedocs.io/en/latest/visual_coding_neuropixels.html. Zebrafish: http://www.nature.com/articles/nmeth.2434. Biolocomotion: http://github.com/meganebers/mobileSHRED, http://simtk.org/projects/ankleexopred and [65,66,67].

## Theme

One contribution of 12 to a discussion meeting issue ‘Digital healthcare for the management of functional neurological disorders’.

## Appendix A. Hyperparameter tuning

### (a) Experiment A.I

— *Data:* 1500 time samples for each of the five worms which were trained separately.
— *SR:* 2.8–2.9 Hz depending on the worm.
— *Neurons:* 109–135 neurons depending on the worm.
— *Neurons selected:* three.
  Sensitivity analysis in §3a(iii) shows that three is sufficient to reconstruct population activity.
— *Lag:* 100.
  A suitable lag should balance two factors: (i) it must not be so large that training becomes computationally inefficient and (ii) it must not be so small that important long-range temporal dependencies are missed. There is no precise formula for choosing the lag; one practical approach, as used by the author, is to experiment with different lag values and visually assess which choice appears most reasonable (See figure 15 for examples).
— *Train/Test/Val split:* 80 : 10 : 10 as discussion in §3a(iii) of the manuscript.
— *Latent dimension:* 32.
  The latent dimension should be chosen to be large enough to capture the complexity of the time-series data, but not so large that it leads to excessive training time or reduced interpretability.
— *Number of epochs:* 200.
  This number is large enough to effectively train the model without overfitting. For SINDy-SHRED, around 200 training epochs is generally considered standard.

### (b) Experiment A.II

— *Data:* 1000 for each of the five worms. The data were concatenated together so that worms 1–3 were the training set, worm 4 was the validation set and worm 5 was the test set.
— *SR:* 2.8–2.9 Hz depending on the worm.
— *Neurons:* 45 neurons (recorded from all five worms).
— *Neurons selected:* three.
  Sensitivity analysis in §3a(iii) shows that three is sufficient to reconstruct population activity.
— *Lag:* 100.
  A suitable lag should balance two factors: (i) it must not be so large that training becomes computationally inefficient and (ii) it must not be so small that important long-range temporal dependencies are missed. There is no precise formula for choosing the lag; one practical approach, as used by the author, is to experiment with different lag values and visually assess which choice appears most reasonable (see figure 15).
— *Train/Test/Val split:* Manually set so that worms 1–3 = train, worm 4 = validation and worm 5 = test.
— *Latent dimension:* 32.
  The latent dimension should be chosen to be large enough to capture the complexity of the time-series data, but not so large that it leads to excessive training time or reduced interpretability.
— *Number of epochs:* 200.
  This number is large enough to effectively train the model without overfitting. For SINDy-SHRED, around 200 training epochs is generally considered standard.

### (c) Experiment A.III

The goal of experiment A.III was to examine how the number of neurons, the train/test split and variance-based preprocessing influence SHRED's performance. Such sensitivity analyses are useful for identifying reasonable choices for the hyperparameters described above.

### (d) Experiment B.I

— *Data:* 1000 for each stimulus presentation. The three presentations were concatenated to create one data matrix. The first two presentations served as the training dataset while the third presentation was split into the testing and validation set.
— *SR:* 500 Hz for the DG stimulus (2-s duration) and 4 kHz for the flash stimulus (0.25 s). The SRs are different since the duration of the stimuli differs.
— *Sensors:* 74 for probe A and 76 for probe C (see figure 16).
— *Sensors selected:* three.
  Sensitivity analysis in §3a(iii) shows that three sensors are sufficient to reconstruct population activity.
— *Lag:* 100.
  A suitable lag should balance two factors: (i) it must not be so large that training becomes computationally inefficient and (ii) it must not be so small that important long-range temporal dependencies are missed. There is no precise formula for choosing the lag; one practical approach, as used by the author, is to experiment with different lag values and visually assess which choice appears most reasonable. Figure 16 is a heatmap showing 100 time points. For this dataset, it seems that 100 is sufficient to capture some temporal dynamics, thus was selected to be the lag for these experiments.
— *Train/Test/Val split:* The first two stimulus presentations were assigned to the training set. The last presentation was split into the testing and validation sets.
— *Latent dimension:* 32.
  The latent dimension should be chosen to be large enough to capture the complexity of the time-series data, but not so large that it leads to excessive training time or reduced interpretability.
— Both a latent dimension of 32 and 64 were tested to compare accuracy. A latent dimension of 32 produced better results; thus, the authors used a dimension of 32 for the rest of the experiments in part B.I.
— *Number of epochs:* 200.
  This number is large enough to effectively train the model without overfitting. For SINDy-SHRED, around 200 training epochs is generally considered standard.

### (e) Experiment B.II

— *Data:* 1000 for each stimulus presentation.
— *SR:* 500 Hz for the DG stimulus (2-s duration) and 4 kHz for the flash stimulus (0.25 s). The SRs are different since the duration of the stimuli differs.
— *Neurons:* 243 units selected with SNR > 3.
— *Neurons selected:* three.
  Sensitivity analysis in §3a(iii) shows that three sensors are sufficient to reconstruct population activity.
— *Lag:* 100.
  A suitable lag should balance two factors: (i) it must not be so large that training becomes computationally inefficient and (ii) it must not be so small that important long-range temporal dependencies are missed. There is no precise formula for choosing the lag; one practical approach, as used by the author, is to experiment with different lag values and visually assess which choice appears most reasonable. For this dataset, it seems that 100 is sufficient to capture some temporal dynamics; thus, was selected to be the lag for these experiments.
— *Train/Test/Val split*: 80 : 10 : 10.
— *Latent dimension*: 64.
  The latent dimension should be chosen to be large enough to capture the complexity of the time-series data, but not so large that it leads to excessive training time or reduced interpretability.
Both a latent dimension of 32 and 64 were tested to compare accuracy. Since this dataset contains very high-frequency fluctuations, a larger latent dimension produced better results. Thus, a latent dim of 64 was used for both the flashes and the DG responses.
— *Number of epochs:* 200.
  This number is large enough to effectively train the model without overfitting. For SINDy-SHRED, around 200 training epochs is generally considered standard.

### (f) Experiment B.III

— *Data:* 2502 time points for firing rate and LFP.
— *SR:* 1.25 kHz.
— *Sensors:* two (firing rate and nearby LFP).
— *Neurons selected:* Firing rate.
  The goal of this experiment was to reconstruct LFP from the firing rate.
— *Lag:* 100.
  A suitable lag should balance two factors: (i) it must not be so large that training becomes computationally inefficient and (ii) it must not be so small that important long-range temporal dependencies are missed. There is no precise formula for choosing the lag; one practical approach, as used by the author, is to experiment with different lag values and visually assess which choice appears most reasonable.
— *Train/Test/Val split:* 80 : 10 : 10.
— *Latent dimension:* 64.
  The latent dimension should be chosen to be large enough to capture the complexity of the time-series data, but not so large that it leads to excessive training time or reduced interpretability.
  Both a latent dimension of 32 and 64 were tested.
— *Number of epochs:* 200.
  This number is large enough to effectively train the model without overfitting. For SINDy-SHRED, around 200 training epochs is generally considered standard.

### (g) Experiment B.IV

— *Data:* 1200 time points of probe A LFP recording in response to the DG stimulus and corresponding pupil area recordings.
— *SR:* 600 Hz.
— *Sensors:* One pupil area trace and 74 channels for LFP recordings.
— *Neurons selected:* Pupil area.
  The aim of this experiment was to reconstruct LFP from just pupil area.
— *Lag:* 100.
  A suitable lag should balance two factors: (i) it must not be so large that training becomes computationally inefficient and (ii) it must not be so small that important long-range temporal dependencies are missed. There is no precise formula for choosing the lag; one practical approach, as used by the author, is to experiment with different lag values and visually assess which choice appears most reasonable.
— *Train/Test/Val split:* 80 : 10 : 10.
— *Latent dimension:* 32.
  The latent dimension should be chosen to be large enough to capture the complexity of the time-series data, but not so large that it leads to excessive training time or reduced interpretability.
  A latent dimension of 16 was also tested, but was too small to reconstruct even the smaller scale patterns in LFP.
— *Number of epochs:* 200.
  This number is large enough to effectively train the model without overfitting. For SINDy-SHRED, around 200 training epochs is generally considered standard.

### (h) Experiment C.I

— *Data:* Whole-brain imaging of neuronal activity (visualization C, raw ΔF/F) found in the supplementary information section of http://www.nature.com/articles/nmeth.2434#Sec20. The original video (forebrain.avi) was cropped to only include the left-hand side which shows the dorsal maximum-intensity projections of whole-brain functional activity and maximum-intensity projections of the reference anatomy (grey). 750 time points were obtained.
— *SR:* 0.72 Hz.
— *Dimensions:* 750 (time) × 472 × 600 → 750 × 283 200 pixels. 
— *Neurons selected:* 250.
  Previous experiments have shown that three neurons are sufficient as input to SHRED. However, so far, the total number of channels was 135 or less. Thus, in the previous experiments, roughly 2% of the sensors were selected. In this example, we have significantly more sensors than before. Even selecting 300 pixels is still less than 0.1% of the total number of sensors.
— *Lag:* 100.
  A suitable lag should balance two factors: (i) it must not be so large that training becomes computationally inefficient and (ii) it must not be so small that important long-range temporal dependencies are missed. There is no precise formula for choosing the lag; one practical approach, as used by the author, is to experiment with different lag values and visually assess which choice appears most reasonable.
— *Train/Test/Val split:* 80: 10 : 10.
— *Latent dimension:* 128.
  The latent dimension should be chosen to be large enough to capture the complexity of the time-series data, but not so large that it leads to excessive training time or reduced interpretability. A slightly higher dimension was chosen for this experiment to account for the increased complexity of the dataset.
— *Number of epochs:* 200.
  This number is large enough to effectively train the model without overfitting. For SINDy-SHRED, around 200 training epochs is generally considered standard.
